# Sociodemographic characteristics and COVID-19 testing rates: spatiotemporal patterns and impact of test accessibility in Sweden

**DOI:** 10.1093/eurpub/ckad209

**Published:** 2023-11-27

**Authors:** Beatrice Kennedy, Georgios Varotsis, Ulf Hammar, Diem Nguyen, Germán D Carrasquilla, Vera van Zoest, Robert S Kristiansson, Hugo Fitipaldi, Koen F Dekkers, Meena Daivadanam, Mats Martinell, Jonas Björk, Tove Fall

**Affiliations:** Department of Medical Sciences, Molecular Epidemiology and Science for Life Laboratory, Uppsala University, Uppsala, Sweden; Department of Medical Sciences, Molecular Epidemiology and Science for Life Laboratory, Uppsala University, Uppsala, Sweden; Department of Medical Sciences, Molecular Epidemiology and Science for Life Laboratory, Uppsala University, Uppsala, Sweden; Department of Medical Sciences, Molecular Epidemiology and Science for Life Laboratory, Uppsala University, Uppsala, Sweden; Novo Nordisk Foundation Center for Basic Metabolic Research, Faculty of Health and Medical Sciences, University of Copenhagen, Copenhagen, Denmark; Department of Information Technology, Division of Systems and Control, Uppsala University, Uppsala, Sweden; Department of Systems Science for Defence and Security, Swedish Defence University, Stockholm, Sweden; Department of Public Health and Caring Sciences, Uppsala University, Uppsala, Sweden; Diabetic Complications Unit, Department of Clinical Sciences, Lund University Diabetes Centre, Lund, Sweden; Department of Medical Sciences, Molecular Epidemiology and Science for Life Laboratory, Uppsala University, Uppsala, Sweden; Global Health and Migration Unit, Department of Women’s and Children’s Health, Uppsala University, Uppsala, Sweden; Department of Public Health and Caring Sciences, Uppsala University, Uppsala, Sweden; Division of Occupational and Environmental Medicine, Lund University, Lund, Sweden; Clinical Studies Sweden, Forum South, Skåne University Hospital, Lund, Sweden; Department of Medical Sciences, Molecular Epidemiology and Science for Life Laboratory, Uppsala University, Uppsala, Sweden

## Abstract

**Background:**

Diagnostic testing is essential for disease surveillance and test–trace–isolate efforts. We aimed to investigate if residential area sociodemographic characteristics and test accessibility were associated with Coronavirus Disease 2019 (COVID-19) testing rates.

**Methods:**

We included 426 224 patient-initiated COVID-19 polymerase chain reaction tests from Uppsala County in Sweden from 24 June 2020 to 9 February 2022. Using Poisson regression analyses, we investigated if postal code area Care Need Index (CNI; median 1.0, IQR 0.8–1.4), a composite measure of sociodemographic factors used in Sweden to allocate primary healthcare resources, was associated with COVID-19 daily testing rates after adjustments for community transmission. We assessed if the distance to testing station influenced testing, and performed a difference-in-difference-analysis of a new testing station targeting a disadvantaged neighbourhood.

**Results:**

We observed that CNI, i.e. primary healthcare need, was negatively associated with COVID-19 testing rates in inhabitants 5–69 years. More pronounced differences were noted across younger age groups and in Uppsala City, with test rate ratios in children (5–14 years) ranging from 0.56 (95% CI 0.47–0.67) to 0.87 (95% CI 0.80–0.93) across three pandemic waves. Longer distance to the nearest testing station was linked to lower testing rates, e.g. every additional 10 km was associated with a 10–18% decrease in inhabitants 15–29 years in Uppsala County. The opening of the targeted testing station was associated with increased testing, including twice as high testing rates in individuals aged 70–105, supporting an intervention effect.

**Conclusions:**

Ensuring accessible testing across all residential areas constitutes a promising tool to decrease inequalities in testing.

## Introduction

Coronavirus Disease 2019 (COVID-19) diagnostic testing has been a cornerstone in the early detection of cluster outbreaks and variants of concern, disease surveillance, population-based assessments of vaccine effectiveness and the evaluation of policy effects.^1^ Sufficient testing was also essential for successful test–trace–isolate programmes, which helped break the chains of COVID-19 transmission.^2^^,^^3^ However, COVID-19 testing rates varied considerably even within countries with uniform testing strategies, with several studies noting low COVID-19 testing in disadvantaged communities.^4–10^ In Sweden, previous studies have reported associations between sociodemographic circumstances and COVID-19 morbidity and mortality.^11^^,^^12^ Further, COVID-19 test uptake has also been linked to access to testing stations and/or mobile test units.^9^^,^^13^ Elucidating the sociodemographic and geographical determinants of COVID-19 testing may improve testing strategies to successfully reach all inhabitants.

In accordance with national guidelines from the Swedish Public Health Agency, free-of-charge population-based COVID-19 polymerase chain reaction (PCR) diagnostic testing was available in Sweden between June 2020 and February 2022. In Uppsala County (total population of 386 000), which includes Sweden’s fourth most populated city Uppsala, the healthcare council initially established four testing stations for patient-initiated tests in June 2020. When case notification rates surged in October 2020, additional testing stations were opened, and a mobile testing unit was deployed to potential hotspots. The testing strategy was subsequently updated to include drop-in testing and self-sampling kits.

We aimed to investigate the associations between sociodemographic characteristics of residential areas and COVID-19 testing rates in Uppsala County and Uppsala City, from June 2020 to February 2022. Such findings could help explain the previously observed sociodemographic differences in COVID-19 outcomes, and provide guidance for future targeted interventions in disadvantaged communities.

## Methods

### Postal code areas

Sweden is divided into 5-digit postal code areas, of which 533 postal code areas overlap with Uppsala County (Swedish: ‘Region Uppsala’). We excluded 172 postal code areas that were uninhabited, 10 that primarily overlapped with neighbouring counties and 1 for which the Care Need Index (CNI) had not been calculated (<3 residents). We categorized the remaining 350 postal code areas as Uppsala City (postal codes beginning with 75; *n* = 147) and Uppsala County (74 or 81; *n* = 203).

### Care Need Index

We obtained data on the CNI from 2020 for each postal code area from Statistics Sweden. CNI constitutes a composite measure computed using seven demographic and socioeconomic variables, i.e. proportion of inhabitants who are (i) <5 years, (ii) born in eastern Europe (outside the European Union), Asia, Africa or South America, (iii) >65 years and reside in single-person households, (iv) single parents with children <18 years, (v) >1 year and have moved into the postal code area within the previous calendar year, (vi) 25–64 years with low educational attainment (≤9 years of schooling), (vii) 16–64 years and unemployed or enrolled in a labour market programme. CNI is used to allocate primary healthcare resources (see Supplementary data),^14–16^ and higher CNI indicates higher primary healthcare need. Postal code area CNI constituted our main exposure variable.

### Study period

Until June 2020, COVID-19 PCR testing in Sweden was only available to healthcare professionals, nursing home residents and hospital patients.^17^ From 24 June 2020, updated national guidelines recommended free-of-charge PCR testing for all individuals in Sweden aged ≥16 with symptoms of COVID-19. Testing in children aged ≥9 was made available on 1 August 2020, and in children aged ≥5 on 22 February 2021. Large-scale testing was discontinued on 10 February 2022.^18^ Our study period was defined as 24 June 2020–9 February 2022.

### COVID-19 testing

Information on COVID-19 PCR tests [date of test and test result (positive/negative)] was accessed from the electronic medical records database maintained by Uppsala County Council. In total, 631 412 PCR diagnostic tests were performed during the study period in residents of the 350 postal code areas. We excluded tests performed as part of the screening and tracing efforts of non-symptomatic staff and care recipients within healthcare and/or elderly care (*N* = 108 188), tests that were requested by a physician and not patient-initiated (*N* = 96 865), and tests performed in individuals aged <5 or >105 (*N* = 41). We excluded tests in children aged <16 years before 1 August 2020 (*N* = 25) and in children aged <9 years before 22 February 2021 (*N* = 69). The final dataset consisted of 426 224 patient-initiated tests. We did not have access to personal identifiers, and the dataset therefore includes repeated tests from individuals. The aggregated daily number of tests per sex (women/men) and per age group (5–14, 15–29, 30–49, 50–69, 70–105 years) in each postal code area constituted our main outcome.

### COVID-19 case notifications, hospital admissions and vaccinations

We calculated daily COVID-19 case notification rates per 100 000 inhabitants, per sex, age group and postal code area. These case notification rates constituted the main marker of community transmission in our analyses.

We accessed information on daily COVID-19 hospital admissions, defined as patients with a positive COVID-19 PCR test within 30 days prior to admission or during the hospital stay, per 100 000 inhabitants per postal code area. Information per sex and age group was not available. We used hospital admissions as a secondary marker of community transmission.

We extracted information on aggregate vaccination coverage in inhabitants aged 15–105 from Uppsala County Council (see the Supplementary data).

### Test accessibility and interventions

During the first part of the study period (24 June to 11 October 2020), patient-initiated testing was available at the four main testing stations set up across Uppsala County (Supplementary figure S1).

From October 2020, local health authorities introduced several new measures to encourage testing. The Uppsala City neighbourhood Gottsunda exhibited low testing rates but also high proportion of positive tests and high hospital admission rates. Gottsunda was therefore targeted with a testing station that opened 12 October 2020. The new test station was highlighted in a press release, which advertised that the staff was multilingual.^19^

Several new testing stations were subsequently opened across Uppsala County and Uppsala City, and a mobile testing bus was deployed weekly or biweekly to emerging hotspots. For detailed information on testing availability, see the Supplementary data.

### Statistical analysis

We used multivariable Poisson regression analyses with cluster-robust standard errors (where postal code area represented a cluster) to investigate the association between CNI (exposure) and daily number of tests per sex, age group and postal code area (outcome) across the study period (24 June 2020–9 February 2022). The study period for the youngest age groups (5–14 years) started on 1 August 2020 when testing in children was initiated. The natural logarithm (ln) of the population size per sex and age category and postal code was used as an offset in our analysis. Our model results are thus equivalent to modelling test rates.

We adjusted our main models for date, day of week of test (categorical variable Monday–Sunday), sex and age group, Uppsala County/Uppsala City (binary variable), and daily case notification rates per age and sex group per 100 000 inhabitants per postal code area. Interaction terms were added in the model, with 2-, 3-, 4- and 5-fold interactions added between all combinations of date, age group, sex, Uppsala County/Uppsala City and CNI. The purpose of these interaction terms was to allow for CNI to have different associations depending on age, date, city/county and sex. Our approach thus resembles a stratified analysis, but also allows for model-based testing of interaction terms. Case notification rates and dates were modelled using restricted cubic splines (see Supplementary data).

For each pandemic wave captured by our data, we calculated the highest and lowest test rate ratios (TRRs) per sex and age group in Uppsala County and in Uppsala County. As the first pandemic wave in Sweden occurred from March to June 2020, not captured by our data, we have denoted the three waves in our data as the second (7 November 2020–6 January 2021), the third (18 March–6 May 2021) and the fourth wave (31 December 2021–9 February 2022; see Supplementary data).

In a sensitivity analysis, we used daily hospital admission rates per 100 000 inhabitants instead of daily case notification rates as marker of community transmission.

We conducted a separate analysis (24 June to 11 October 2020) with distance to main testing station as main exposure, and daily testing rates as outcome, adjusting for all covariates included in the main analysis as well as for CNI. We determined the driving distance from each postal code area to the nearest test station by Google Maps Distance Matrix API using the R package *gmapsdistance* in R version 4.0.32. The Distance Matrix created a polygon of each postal code area, and the centre of the polygon defined location. The test stations were defined by their street addresses.

To assess any potential intervention effect of the first targeted testing station in the neighbourhood Gottsunda, which opened on 12 October 2020, we further performed a difference-in-difference-analysis on neighbourhood level comparing testing rates in Gottsunda and Sävja (encompassing seven and two postal code areas, respectively) from 14 July 2020 to 10 January 2021.

Gottsunda and Sävja comprised two out of four main disadvantaged areas in Uppsala City. The other two were both targeted with a mobile test station for some weeks in November or December 2020 and were therefore not suitable as control groups. Sävja was not targeted by any specific interventions during this period and was selected as the control area. We employed an adjusted Poisson model with a binary variable for Sävja/Gottsunda (see the Supplementary data), and we performed a sensitivity analysis where we adjusted for daily case notification rates per 100 000.

Finally, we generated graphs of model-based testing rates per 100 000 per sex, age category and Uppsala city/county by setting day of the week to Wednesday and daily case notification rates (or, in the sensitivity analysis, hospital admissions rates) to zero. We used Uppsala County and Uppsala City-specific CNI 10th and 90th percentiles. Similar graphs were constructed with distance to main testing station as the exposure, as well as for the difference-in-difference-analysis on Gottsunda/Sävja.

All analyses were conducted using Stata 15.^10^ Maps and plots were produced using R software (v. 4.1.3, March 2022).^20^ The ‘tmap’ package (v. 3.3.3),^21^ ‘ggplot2’ package (v. 3.3.6),^22^ the ‘ggpattern’ (v. 0.4.2)^23^ and the ‘fontawesome’ package (v. 0.4.0)^24^ were used to produce the maps and plots.

### Ethical statement

This study was approved by the Swedish Ethical Review Authority (DNR 2020-04210 and DNR 2021-01915).

## Results

### Baseline characteristics

**Figure 1.**
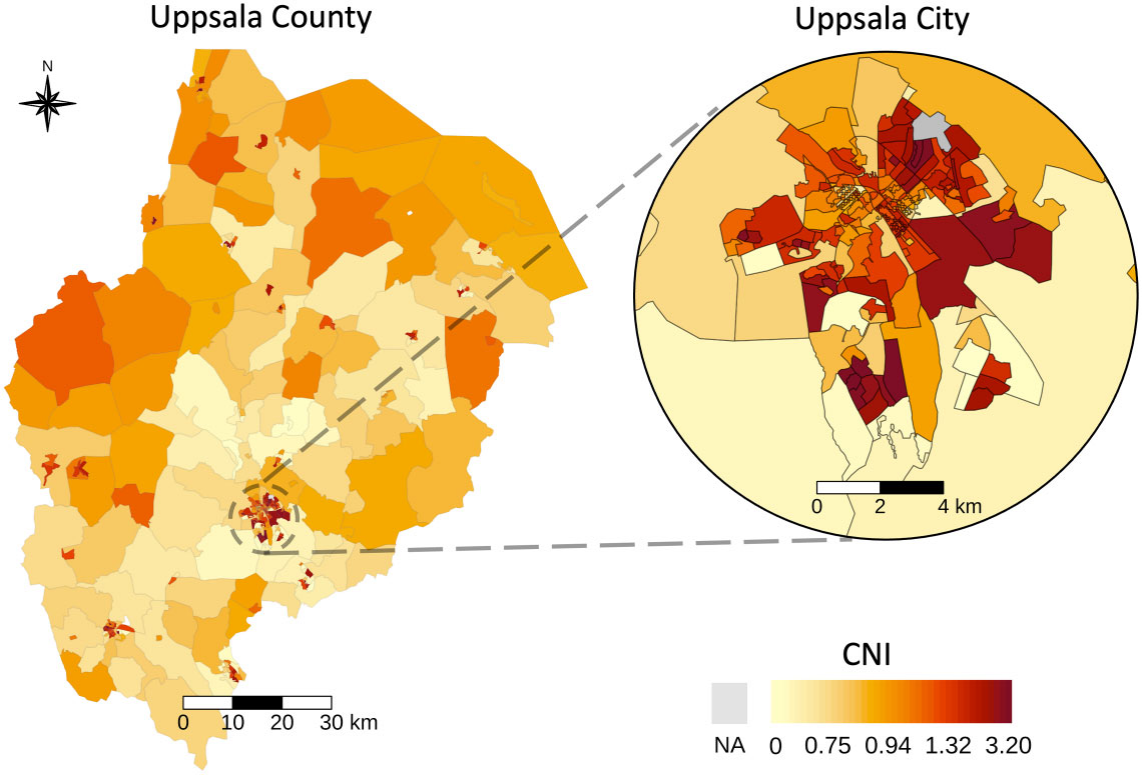
Geographical distribution of Care Need Index (CNI) across postal code areas in Uppsala County and Uppsala City. Higher CNI indicates higher primary health care burden. The colour legend changes non-linearly to facilitate visualization of the skewed distribution.

Baseline characteristics of the 350 postal code areas in Uppsala County and Uppsala City are presented in figure 1 and Supplementary table S1. CNI across all postal code areas ranged from 0.0–3.2 (Supplementary figure S2), with a lower median noted in Uppsala County (0.8, IQR: 0.7–1.1) than in Uppsala City (1.1, IQR: 0.8–1.6). We observed that CNI in both Uppsala County and Uppsala City were strongly correlated with proportion of inhabitants who had compulsory education only or were born in areas outside of the European Union (Supplementary figure S3). The two Uppsala City neighbourhoods Gottsunda and Sävja had higher CNI than the Uppsala City average (Supplementary table S2).

### COVID-19 testing, case notification and hospital admission rates

Testing and case notifications rates demonstrated similar patterns in inhabitants from Uppsala County and Uppsala City across the study period, with the three peaks of case notifications coinciding with the three peaks of hospital admissions (Supplementary figure S4). The highest testing rates were observed in Uppsala City (7-day rolling average of >400 tests per 100 000) in April and December 2021. Inhabitants aged 70–105 had the overall lowest testing rates across age groups (Supplementary figure S5).

### COVID-19 vaccination coverage

The population-weighted median cumulative vaccination coverage in inhabitants aged 15–105 years reached 87.9% (IQR 84.7, 90.1) in Uppsala County and 88.5% (IQR 83.3, 90.6) in Uppsala City on 9 February 2022. Across CNI quartiles, we observed an inverse association between CNI and vaccination coverage (Supplementary figure S6), with vaccination rates ranging from 84.3% to 90.1% in Uppsala County and from 79.1% to 91.0% in Uppsala City.

### CNI was associated with COVID-19 testing rates

**Figure 2.**
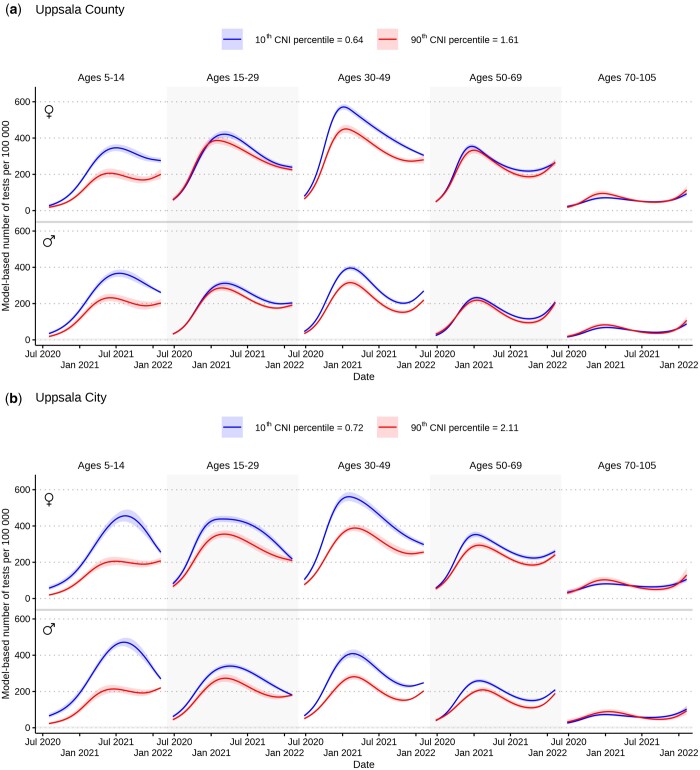
Model-based testing rates for COVID-19 per sex and age group per 100 000 inhabitants in Uppsala County (not including Uppsala City) (**a**) and Uppsala City (**b**) across the study period (24 June 2020–9 February 2022), presented by 10th and 90th postal code area Care Need Index (CNI) percentiles. Higher Care Need Index (CNI) indicates higher primary health care burden.

**Figure 3.**
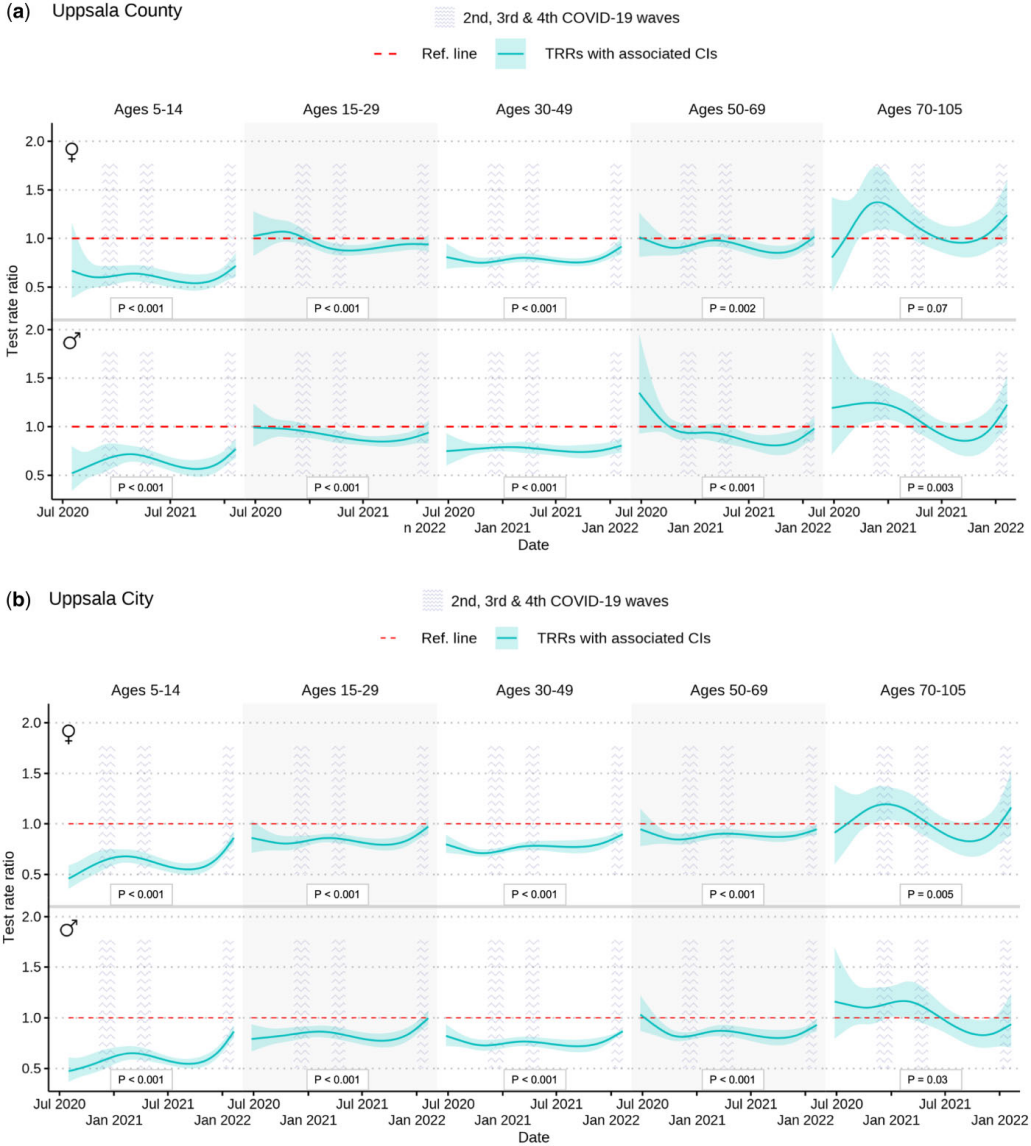
COVID-19 test rate ratios (TRRs) and 95% confidence intervals (CIs) by postal code area Care Need Index (CNI) per sex and age group per 100 000 inhabitants in Uppsala County (not including Uppsala City) (**a**) and Uppsala City (**b**) across the study period (24 June 2020–9 February 2022) and the three pandemic waves captured by our study. A TRR of 0.9 indicates that each unit increase of CNI was associated with a 10% decrease in testing per 100 000 in that sex and age group. CNI across all postal code areas ranged from 0.0 to 3.2, with a median in Uppsala County of 0.8 (IQR: 0.7, 1.1) and in Uppsala City of 1.1 (IQR: 0.8, 1.6).

The association between CNI and testing rates was investigated by estimating test rate ratios (TRRs). A TRR of 0.9 indicated that each unit increase of CNI was associated with a 10% decrease in testing per 100 000 in that sex and age group. We observed a negative association between CNI and testing rates in several age groups (aged 15–69) in both women and men (figures 2 and 3, Supplementary figure S7). These patterns were generally more pronounced in Uppsala City than in Uppsala County, e.g. TRRs from Uppsala City ranged from 0.71 (95% CI 0.68–0.75) to 0.90 (95% CI 0.85–0.94) in individuals aged 30–49 across the three separate pandemic waves (Supplementary table S3). Further, CNI was consistently negatively associated with testing rates in children aged 5–14 across all three pandemic waves in both Uppsala City and Uppsala County, with TTRs ranging from 0.56 (95% CI 0.47–0.67) to 0.87 (95% CI 0.80–0.93).

In contrast, in women aged 70–105 in Uppsala County and Uppsala City, and in men aged 70–105 in Uppsala County, CNI was positively associated with testing rates during the second pandemic wave (7 November 2020–6 January 2021), with TRRs ranging from 1.17 (95% CI 1.01–1.36) to 1.37 (95% CI 1.09–1.74). We could not detect any similar associations in these age groups during subsequent waves.

Our sensitivity analysis, where we employed daily hospital admissions as a marker of community transmission, yielded similar results as the main model (Supplementary figure S8, Supplementary table S4).

### Distance to testing station and the intervention effect

From 24 June to 11 October 2020, the median distance to main testing station was 24.2 and 4.1 km for postal code areas in Uppsala County and Uppsala City, respectively. We found that longer distance to testing stations was independently associated with lower testing rates after adjusting for CNI. The association was overall most prominent in younger age groups (9–14 and 15–29 years) in Uppsala County (Supplementary figure S9, Supplementary tables S5 and S6), and in children aged 9–14 years in Uppsala City, e.g. we observed TRRs in inhabitants aged 15–29 years in Uppsala County ranging from 0.815 (95% CI 0.735–0.904) to 0.901 (95% CI 0.847–0.959). Here, a TRR of 0.901 indicates that each additional 10 km was associated with a 9.9% decrease in testing per 100 000.

**Figure 4.**
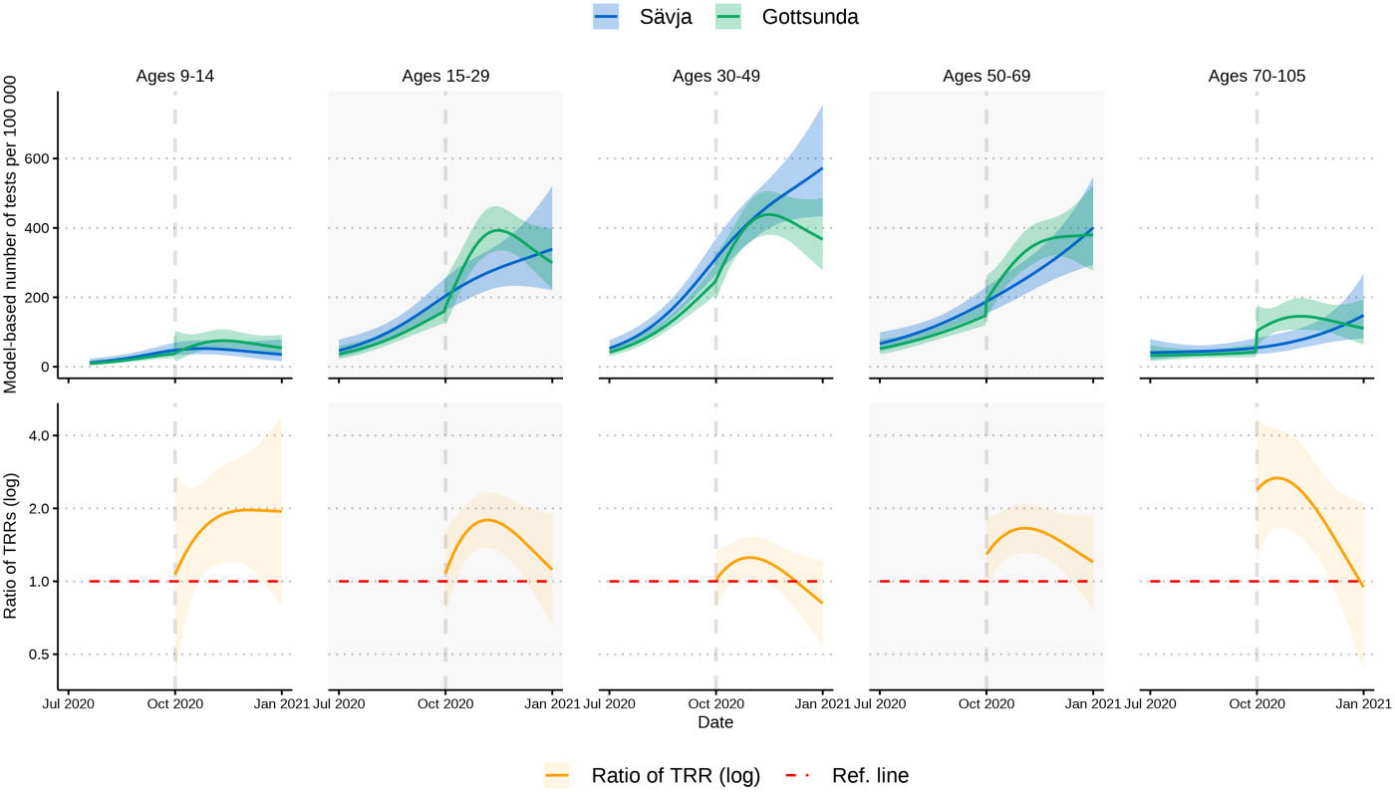
COVID-19 testing in the two Uppsala City neighbourhoods Gottsunda and Sävja 14 July 2020–10 January 2021, with Gottsunda targeted by local health authorities with a local testing station opened on 12 October 2020. Top panels depict model-based testing rates per age group per 100 000 inhabitants in Gottsunda and Sävja. Bottom panels depict results from difference-in-difference-analysis [ratio of test rate ratios (TRRs)] comparing Gottsunda and Sävja per age group. Higher ratio of TRRs indicates relative increase of testing in Gottsunda (omnibus *P* value: <0.001).

We further observed that the opening of a targeted testing station in Gottsunda on 12 October 2020 coincided with increased testing rates, as compared with the non-targeted neighbourhood Sävja (figure 4, Supplementary figures S10 and S11). The difference-in-difference-analysis indicated an overall intervention effect (omnibus *P* values: <0.001). We observed transient differences in testing rates across all age groups 15–105 years, with initially more than twice as high testing rates in the oldest group (70–105 years). We also noted increased testing over time in children aged 9–14. In total, 1888 patient-initiated tests were conducted in inhabitants 9–105 years in Gottsunda from 12 October 2020 to 10 January 2021. We estimated that 582 out of these tests were attributable to the intervention. In the sensitivity analysis where we adjusted our intervention model for daily case notification rates, the number of attributable tests was 410.

## Discussion

We found that postal code area CNI was negatively associated with both adult and child testing COVID-19 rates across the study period, which encompassed three separate pandemic waves. Longer distance to the nearest testing station was associated with lower testing rates, more pronounced in Uppsala County that included less densely populated areas. The opening of a new test station in a disadvantaged neighbourhood with high community transmission rates was linked to an increase in testing.

Our findings align with a report from the Skåne healthcare region in Sweden using data from June 2020 to April 2021,^25^ with research from the USA and the UK,^5–9^ as well as with Swiss studies comprising data on COVID-19 testing rates, hospitalizations and deaths in 2020–21.^4^^,^^10^ A study investigating COVID-19 mortality in Sweden found that many migrant groups were particularly vulnerable during the first pandemic wave, and that part of the excess risk could be attributed to demographic factors such as neighbourhood population density.^12^ The importance of neighbourhood characteristics, independent of individual-level risk factors, was also highlighted in a later report from the Stockholm County Council on COVID-19 mortality, indicating an interplay between individual risk factors and residential area circumstances.^11^ As our study does not include individual-level data, we cannot distinguish the influence of residential- and individual-level factors on testing, and both likely contributed to our findings. At the end of the study period, we observed that CNI was also inversely associated with vaccination coverage.

Overall, we noted lower patient-initiated test rates in individuals 70–105 years than in younger individuals. This corresponds with previous findings for Sweden where test rates were considerably lower in individuals ≥65 years than in those aged 20–64.^3^ In addition, we excluded tests performed in screening and tracing efforts within healthcare and/or elderly care as well as physician-initiated tests where a higher proportion of tests were conducted in the elderly. Primary health care centres however performed a very limited number of tests during our study period, and the lower patient-initiated test rates in the oldest age group observed in the present study cannot be explained by increased contact with their general practitioner.

We observed that during the second pandemic wave, testing rates were higher in inhabitants aged 70–105 years residing in areas with higher CNI than in those residing in areas with lower CNI, a pattern not noted during subsequent waves. It is possible that older age groups residing in more affluent neighbourhoods could more easily adhere to the 2020 national non-mandatory COVID-19 recommendations of physical distancing for individuals aged ≥70.^26^ This would correspond to a Norwegian COVID-19 study that reported a positive association between individual-level income and education level and adherence to non-pharmaceutical interventions such as physical distancing and avoidance of public transportation.^27^

We observed that longer distances to the nearest testing station were associated with lower testing rates in younger age groups, even after adjusting for CNI. This finding is consistent with an English study on spatial inequalities in large-scale COVID-19 antigen testing,^9^ as well with other previous studies that have indicated worse health outcomes and lower adherence to health prevention and screening programmes among individuals living farther away from healthcare facilities, compared with those who lived closer.^28^^,^^29^ Our findings indicate that future successful COVID-19 test efforts should be decentralized, and that access to testing should not require public transport or car ownership. Mobile test units likely constitute valuable additions to enable testing in remote areas or to quickly increase testing capacity in emerging hotspots.

We leveraged a difference-in-difference-analysis, a quasi-experimental design that can employ longitudinal data to estimate causal effects of interventions,^30^ to evaluate the targeting of Gottsunda with a testing station. The opening was accompanied by an information campaign and was highlighted in local media outlets. We observed an intervention effect, with the largest increase in testing in the oldest age groups, which are also the most vulnerable to adverse COVID-19 outcomes.^31^^,^^32^ Our findings emphasize that neighbourhoods with low testing rates would likely benefit from testing interventions tailored to that specific community.

## Strengths and limitations

Strengths of the study include the use of detailed data extracted from population and health registers on postal code area sociodemographic characteristics and COVID-19 testing, case notification and hospital admission rates. All PCR tests conducted in Uppsala County and City were administered and analysed by the Uppsala County Council, ensuring that our study comprises all tests from a Swedish county, which had uniform testing recommendations and booking procedures across the study period. Some potential limitations apply. Firstly, the use of aggregate data on sociodemographic circumstances and on testing, also including repeated tests, entails that contextual and individual effects cannot be disentangled. Secondly, even though we adjusted for markers of community transmission, we cannot exclude the possibility of residual confounding by differences in infection rates not fully captured by our models. Thirdly, we cannot ascertain that our findings are generalizable to other healthcare regions in Sweden with different testing strategies, population compositions and geospatial structures, or to other countries where testing relied more on lateral flow tests and/or preventive screening of asymptomatic individuals. Lastly, we were limited to only one intervention area and one control area in the intervention analysis. This both placed strong assumptions on the control area and resulted in a small sample, which may have made the model more unstable and sensitive to, for example, the number and placement of knots for dates.

## Conclusion

CNI is currently employed by the Swedish health authorities to allocate primary healthcare resources. Our findings highlight that CNI should also be considered a readily available tool for allocating and evaluating COVID-19 and epidemic test strategies. Our results also emphasize how real-time monitoring of testing rates and decentralized testing with targeted efforts may ameliorate inadequate testing rates.

## Supplementary Material

ckad209_Supplementary_Data

## Data Availability

Restrictions apply to the availability of the testing and vaccination data, which were used under license and ethical approval. The data are however available from the authors upon reasonable request and with written permission from the Ethical Review Authority in Sweden. Information on postal code areas included in analyses and the code used in the project are available on Github (https://doi.org/10.5281/zenodo.7919371). Key points Free-of-charge COVID-19 PCR diagnostic testing was available to the public in Uppsala County and Uppsala City, Sweden, from 24 June 2020 to 9 February 2022, in accordance with the national guidelines from the Swedish Public Health Agency. Across the study period, which included three separate pandemic waves, we observed lower COVID-19 testing rates in residential areas with higher Care Need Index, a composite measure of area sociodemographic factors used to allocate primary healthcare resources in Sweden. COVID-19 testing was initially centralized at four main testing stations across Uppsala County and Uppsala City, and during this period, longer distance to the nearest testing station was associated with lower testing rates in younger inhabitants and in less densely populated areas. The opening of a local testing station in a disadvantaged neighbourhood was associated with a subsequent increase in testing rates noted across all ages, and with twice as high testing rates in inhabitants aged 70–105. Ensuring accessible COVID-19 testing across all residential areas may constitute an effective tool for decreasing differences in testing rates.
