# Supplementary material for: Sociodemographic characteristics and COVID-19 testing rates: spatiotemporal patterns and impact of test accessibility in Sweden
Source: Eur J Public Health. 2023 Nov 27;34(1):14–21. doi: 10.1093/eurpub/ckad209 (PMC10843959; doi:10.1093/eurpub/ckad209)
Supplement: ckad209_Supplementary_Data [file ckad209_supplementary_data.pdf]

SUPPLEMENTARY MATERIAL

The Care Need Index and choropleth maps

The composite measure Care Need Index (CNI) is computed using seven demographic and socioeconomic variables, i.e. proportion of inhabitants in a postal code area who are 1) <5 years old, 2) born in eastern Europe (outside the European Union), Asia, Africa, or South America, 3) >65 years and reside in single-person households, 4) single parents with children <18 years old, 5) >1 year old and have moved into the postal code area within the previous calendar year, 6) 25-64 years old with low educational attainment (≤9 years of schooling, equivalent to compulsory education only in Sweden), 7) 16-64 years old and unemployed or enrolled in a labour market programme. The weight of each variable depends on the influence on primary healthcare workload, as determined by individual scoring by a panel of primary healthcare physicians (1-3).

To calculate the Care Need Index (CNI) for each postal code area in Sweden in 2020, Statistics Sweden performed the following sequential steps: To assess CNI per person, the sum of all seven sociodemographic variables, calculated as the number of individuals per variable multiplied by a variable-specific weight, was divided by the total number of inhabitants per postal code area. Then, the CNI per person was divided by the CNI per person for the median CNI postal code area, which was 2.13.

We created choropleth maps of CNI per postal code area across Uppsala County and Uppsala City. We generated the geographic features by using administrative shape files derived from an external company, Postnummerservice Norden AB, containing detailed information on five-digit postal code area boundaries. The resulting geospatial polygons were coloured with light-to-dark sequential schemes to represent the CNI distribution; darker colours represent higher CNI. The colour legend changes non-linearly to facilitate the visualisation of the skewed distribution.

COVID-19 testing availability

When diagnostic COVID-19 testing was made available to the general public in Sweden in the end of June 2020, each of the 21 Swedish healthcare regions developed their separate strategies on how to ensure accessible testing for their populations. In Uppsala County, patient-initiated COVID-19 PCR tests were conducted at for the four main testing stations set up - in the north, east, and northwest part of the county, and in the centre of Uppsala City. Tests were pre-booked through the national online healthcare platform Healthcare Guide 1177.se (hereafter, 1177). All time slots for tests were made available in three-day rolling increments. The online booking required access to electronic identification by BankID, a widespread digital national identification certificate. During our study

period, more than 98% of all Swedish citizens aged 18-65 years had a BankID (https://www.bankid.com/en/om-oss/statistik). Individuals could also call a phone number provided from initial contact with 1177 or a primary care center. Online booking was only available in Swedish, while phone booking was available in both Swedish and English, and interpreters for other languages were available when necessary. Tests for children aged 5-12 were pre-booked by their legal guardians. For children aged 13-16, the child could pre-book their own test if they had a FrejaID (also a national identification certificate), if not, their parents booked tests by phone. All testing was performed by medical personnel who conducted nasopharyngeal and oropharyngeal swabs. No self-sampling kits were distributed and no drop-in testing was available during this time. Asymptomatic individuals were not declined testing.

A dedicated testing station was established in Gottsunda on 12 October 2020, and was the first additional testing station opened by the local health authorities. Between October 2020 and April 2021, six more testing stations were opened across Uppsala County (in the municipalities of Håbo, Knivsta, Heby and Älvkarleby) and Uppsala City (in the neighbourhoods Löten and Stenhagen). The testing capacity at the local primary care unit in the municipality of Knivsta was also expanded. Between 16 November 2020 and 12 February 2021, a bus serving as a mobile test unit was deployed weekly or biweekly to six emerging hotspots characterized by low testing rates, but high test positivity, within Uppsala City and across Uppsala County. During the winter of 2020–2021, drop-in testing was also initiated at several testing stations in Uppsala County and Uppsala City.

On November 29, 2021, the regional testing strategy transitioned from one focused on health-care administered tests to one focused on self-test PCR kits, which could be collected at the testing stations without prior appointment, registered through 1177 and then dropped off at any testing station for analyses. However, due to extremely high demands for these self-test kits in Uppsala City, the collection of a self-test kit required pre-booking at 1177 from 15 December 2021. COVID-19 PCR testing of the general public in Uppsala County and Uppsala City was discontinued on 10 February 2022 following updates of national guidelines. At this time, Sweden was in the throes of the fourth pandemic wave caused by the variant of concern Omicron, and the previous testing strategy was deemed inefficient (4).

During the entire study period, the Uppsala County Council initiated several communication and media campaigns related to COVID-19 testing, including information about COVID-19 symptoms and the importance of social distancing. The opening of new test stations and deployment of the mobile test unit were announced during news conferences.

COVID-19 vaccinations

The COVID-19 vaccination programme was initiated in Uppsala County and Uppsala City in January 2021. At the end of our study period, all inhabitants ≥12 years had been invited for vaccination, as well as a small number of children aged 5-11 with severe chronic medical conditions (5, 6). We obtained aggregate data on vaccine coverage from the Uppsala County Council, and calculated population-weighted time-updated cumulative vaccine coverage (defined as ≥2 doses) in inhabitants 15-105 years across postal code areas by CNI quartiles. In accordance with the legal regulations of healthcare in Sweden, residents of Uppsala County and Uppsala City were eligible to receive COVID-19 vaccinations in other parts of Sweden. However, we did not have access to vaccination data from other parts of the country in our study.

Pandemic waves

We defined the start of a pandemic wave as when the 14-day cumulative case notification rates (assessed in Uppsala County and Uppsala City combined) exceeded 500 cases per 100 000, and the end of a pandemic wave when the case notification rates dropped below the same threshold, or when the study period ended, whichever came first.

Restricted cubic splines for case notification rates, dates, and hospital admissions

Knots for splines of case notification rates per 100 000 were placed at -10, 500, 20 00 and 40 000. Knots were chosen to fit the distribution of case notification rates, and the knots outside the variable range were added for increased flexibility. For the splines of dates, three knots were placed at time points representing major changes in overall COVID-19 testing strategy: 12 October 2020, when the Gottsunda testing station was opened and centralized testing thus abandoned; 22 February 2021, when the testing of children ≥5 years was initiated; and 13 December 2021, when the testing strategy shifted from assisted PCR tests towards self-sampling PCR kits. Two additional knots were placed in the beginning (13 August 2020) and towards the end of the study period (15 January 2022) to improve statistical modelling. Hospital admission rates were modelled using restricted cubic splines, with knots placed at -10, 35, 75, and 200 to fit the distribution of the admissions.

Difference-in-difference analysis comparing Gottsunda and Sävja

A difference-in-difference analysis constitutes a statistical method to compare outcomes across two groups before and after an intervention (natural or otherwise), that only affects one of the groups (7). The intervention effect is derived by comparing differences estimated by regression models, in our case, the difference in testing rates after versus before the intervention in Gottsunda, and the

difference in testing rates after versus before the date of the intervention (when the Gottsunda station opened) in Sävja.

The neighbourhood Gottsunda comprised the 7 postal code areas 75645, 75649, 75650, 75654, 75656, 75657, and 75658, and the neighbourhood Sävja the 2 postal code areas 75754 and 75755. The difference-in-difference analysis was conducted on the neighbourhood level. We included test data from 180 days, i.e., from 90 days before the intervention to 90 days thereafter (14 July, 2020 to 10 January 2021). The outcome was defined as daily number of tests per age group and neighbourhood (Sävja or Gottsunda). We employed a Poisson model with regular robust standard errors and adjusted for the date, day of week of test, age group, sex and a binary variable for Sävja/Gottsunda. A binary intervention variable was used as exposure. Interaction terms for intervention, age group, and date were added in the model, whereby two- and three-way interaction terms were added between all combinations of the variables. We did not include an interaction for sex due to the low absolute number of tests in the youngest and oldest age groups. Dates were modelled with restricted cubic splines and knots placed at the 10th, 50th, and 90th percentile (1 August, 12 October, 23 December, 2020). To assess the intervention effect, we performed a post-hoc omnibus test of the main effect and all two- and three-way interaction terms involving the intervention variable. We also performed a sensitivity analysis where we adjusted for daily case notification rates per 100,000 (with knots placed in correspondence with the main analysis of CNI and testing rates).

SUPPLEMENTARY TABLES

Supplementary Table 1. Baseline postal code area characteristics in Uppsala County and Uppsala City, weighted for total population per postal code area. Uppsala County comprise postal codes beginning with 74 and 81, and Uppsala City beginning with 75. Values are presented as median (first and third quartiles), unless stated otherwise.

	**Total**	**Uppsala County**	**Uppsala City**
Postal code areas	N=350	N=203	N=147
Care Need Index	1.0 (0.8, 1.4)	0.8 (0.7, 1.1)	1.1 (0.8, 1.6)
Proportion of women, %	49.9 (48.3, 51.6)	49.0 (47.6, 50.2)	51.1 (49.1, 52.7)
Proportion of inhabitants <5 years[ǂ], %	5.6 (4.2, 6.6)	5.8 (5.0, 6.8)	5.1 (3.9, 6.3)
Proportion of inhabitants born in east- or south Europe (outside the European Union), Africa, Asia or South America[ǂ], %	6.2 (3.5, 14.4)	3.9 (2.2, 6.6)	10.5 (5.7, 19.8)
Proportion of inhabitants >65 years who reside in single-person households[ǂ], %	38.3 (29.7, 49.3)	34.3 (28.4, 42.9)	43.7 (31.6, 53.2)
Proportion of inhabitants who are single parents with children <18 years[ǂ], %	2.6 (2.0, 3.2)	2.7 (2.1, 3.3)	2.3 (1.8, 3.2)
Proportion of inhabitants >1 years who moved into area within the previous calendar year[ǂ], %	8.2 (6.2, 11.2)	6.8 (5.3, 8.5)	10.0 (7.7, 13.5)
Proportion of inhabitants with compulsory education only[1ǂ], %	8.8 (5.1, 12.8)	11.5 (8.0, 13.9)	5.5 (3.1, 10.8)
Proportion of inhabitants unemployed or enrolled in labour market measures[2ǂ], %	10.7 (8.0, 16.4)	10.7 (8.0, 14.4)	10.8 (8.1, 17.7)
Distance to the nearest testing station[3], km	7.5 (3.3, 24.3)	24.2 (11.6, 33.9)	4.1 (2.6, 7.2)

[ǂ] Sociodemographic variable included in the composite measure Care Need Index.
[1] In the population aged 25–64 years
[2] In the population aged 16–64 years
[3] Only applicable 24 June–11 October 2020

Supplementary Table 2. Baseline postal code area characteristics for the two Uppsala City neighbourhoods, Gottsunda and Sävja, weighted for total population per postal code area. Values are presented as median (first and third quartiles) unless stated otherwise.

	**Gottsunda**	**Sävja**
Postal code areas	N=7	N=2
Care Need Index	2.8 (2.1, 2.8)	1.4 (1.4, 1.8)
Proportion of women, %	47.2 (46.8, 51.0)	52.7 (50.4, 52.7)
Proportion of inhabitants <5 years[ǂ], %	8.5 (5.8, 9.6)	6.0 (5.7, 6.0)
Proportion of inhabitants born in east- or south Europe (outside the European Union), Africa, Asia or South America[ǂ], %	48.5 (32.3, 49.3)	19.4 (19.4, 28.1)
Proportion of inhabitants >65 years who reside in single-person households[ǂ], %	56.1 (52.9, 59.4)	36.5 (36.5, 42.9)
Proportion of inhabitants who are single parents with children <18 years[ǂ], %	4.6 (3.8, 5.0)	3.7 (3.7, 3.9)
Proportion of inhabitants >1 years who moved into area within the previous calendar year[ǂ], %	9.3 (8.7, 12.2)	8.8 (8.8, 9.3)
Proportion of inhabitants with compulsory education only[1ǂ], %	22.8 (16.6, 26.3)	10.9 (10.9, 17.3)
Proportion of inhabitants who are unemployed or enrolled in labour market measures[2ǂ], %	36.6 (23.4, 39.8)	17.6 (17.6, 20.4)
Distance to the nearest testing station[3], km	5.7 (1.8, 6.9)	8.7 (8.7, 9.1)

ǂ Sociodemographic variable included in the composite measure Care Need Index.
1 In the population aged 25-64 years
2 In the population aged 16-64 years
3 Only applicable 24 June–11 October 2020

Supplementary Table 3. Main model. Highest and lowest test rate ratios (TRRs) with 95 confidence intervals (CIs) for postal code area Care Need Index (CNI) per sex and age groups in Uppsala County and Uppsala City across the three pandemic waves. This main model is adjusted for date, day of week of test, age group, sex, Uppsala County/Uppsala City, and daily case notification rates per age and sex group per 100 000 per postal code area.

	Second pandemic wave 7 November, 2020 – 6 January, 2021				Third pandemic wave 18 March, 2021 – 6 May, 2021				Fourth pandemic wave 31 December, 2021 – 9 February, 2022			
	Uppsala City		Uppsala County		Uppsala City		Uppsala County		Uppsala City		Uppsala County	
	Highest TRR (95% CI)	Lowest TRR (95% CI)	Highest TRR (95% CI)	Lowest TRR (95% CI)	Highest TRR (95% CI)	Lowest TRR (95% CI)	Highest TRR (95% CI)	Lowest TRR (95% CI)	Highest TRR (95% CI)	Lowest TRR (95% CI)	Highest TRR (95% CI)	Lowest TRR (95% CI)
Women, ages 5-14	0.67 (0.60, 0.75)	0.61 (0.52, 0.73)	0.62 (0.54, 0.72)	0.60 (0.50, 0.72)	0.67 (0.60, 0.74)	0.63 (0.57, 0.70)	0.64 (0.56, 0.72)	0.61 (0.53, 0.70)	0.86 (0.79, 0.94)	0.71 (0.66, 0.76)	0.72 (0.60, 0.86)	0.63 (0.55, 0.71)
Men, ages 5-14	0.62 (0.55, 0.71)	0.56 (0.47, 0.67)	0.70 (0.60, 0.82)	0.65 (0.52, 0.80)	0.65 (0.59, 0.72)	0.62 (0.57, 0.68)	0.71 (0.64, 0.79)	0.67 (0.60, 0.75)	0.87 (0.80, 0.93)	0.71 (0.65, 0.76)	0.77 (0.68, 0.88)	0.67 (0.59, 0.75)
Women, ages 15-29	0.84 (0.79, 0.89)	0.81 (0.74, 0.88)	1.05 (0.96, 1.15)	0.97 (0.90, 1.04)	0.86 (0.82, 0.90)	0.84 (0.80, 0.89)	0.89 (0.83, 0.95)	0.88 (0.82, 0.93)	0.97 (0.91, 1.04)	0.89 (0.83, 0.94)	0.94 (0.88, 1.00)	0.94 (0.86, 1.02)
Men, ages 15-29	0.86 (0.80, 0.92)	0.84 (0.77, 0.91)	0.97 (0.89, 1.06)	0.94 (0.88, 1.01)	0.86 (0.80, 0.92)	0.83 (0.77, 0.89)	0.91 (0.84, 0.98)	0.88 (0.82, 0.94)	1.00 (0.93, 1.07)	0.89 (0.83, 0.96)	0.94 (0.83, 1.07)	0.90 (0.83, 0.98)
Women, ages 30-49	0.74 (0.71, 0.78)	0.71 (0.68, 0.75)	0.78 (0.74, 0.82)	0.75 (0.71, 0.80)	0.78 (0.74, 0.83)	0.78 (0.74, 0.82)	0.80 (0.76, 0.85)	0.79 (0.75, 0.83)	0.90 (0.85, 0.94)	0.84 (0.79, 0.89)	0.92 (0.83, 1.01)	0.84 (0.79, 0.89)
Men, ages 30-49	0.75 (0.70, 0.79)	0.73 (0.68, 0.78)	0.79 (0.75, 0.83)	0.78 (0.74, 0.83)	0.77 (0.72, 0.81)	0.76 (0.71, 0.81)	0.79 (0.73, 0.84)	0.77 (0.72, 0.83)	0.87 (0.84, 0.90)	0.80 (0.76, 0.83)	0.81 (0.73, 0.89)	0.78 (0.71, 0.84)
Women, ages 50-69	0.87 (0.82, 0.92)	0.85 (0.79, 0.90)	0.95 (0.89, 1.01)	0.91 (0.83, 0.98)	0.90 (0.86, 0.95)	0.90 (0.86, 0.95)	0.98 (0.91, 1.05)	0.95 (0.89, 1.02)	0.95 (0.89, 1.01)	0.91 (0.87, 0.95)	1.02 (0.93, 1.11)	0.93 (0.87, 0.99)

Men, ages 50-69	0.84 (0.80, 0.88)	0.81 (0.77, 0.86)	0.95 (0.87, 1.04)	0.94 (0.87, 1.00)	0.87 (0.82, 0.93)	0.86 (0.81, 0.92)	0.93 (0.84, 1.03)	0.90 (0.81, 0.99)	0.93 (0.87, 1.00)	0.86 (0.82, 0.92)	0.98 (0.87, 1.10)	0.89 (0.80, 0.99)
Women, ages 70+	1.19 (1.04, 1.37)	1.17 (1.01, 1.36)	1.37 (1.09, 1.74)	1.33 (1.09, 1.63)	1.10 (0.94, 1.28)	1.00 (0.86, 1.16)	1.17 (1.00, 1.37)	1.07 (0.92, 1.24)	1.16 (0.88, 1.53)	0.99 (0.80, 1.23)	1.24 (0.96, 1.60)	1.11 (0.91, 1.36)
Men, ages 70+	1.15 (1.01, 1.31)	1.11 (0.95, 1.30)	1.24 (1.05, 1.47)	1.22 (1.07, 1.39)	1.15 (0.98, 1.35)	1.08 (0.92, 1.26)	1.12 (0.98, 1.28)	1.02 (0.90, 1.16)	0.94 (0.71, 1.24)	0.87 (0.72, 1.06)	1.23 (0.99, 1.52)	1.04 (0.86, 1.26)

Supplementary Table 4. Sensitivity analysis. Highest and lowest test rate ratios (TRRs) with 95 confidence intervals (CIs) for Care Need Index (CNI) in sex and age groups in Uppsala County and Uppsala City across the three pandemic waves. This sensitivity analysis model is adjusted for date, day of week of test, age group, sex, Uppsala County/Uppsala City, and daily hospital admissions per 100 000 per postal code area.

	Second pandemic wave 7 November, 2020 – 6 January, 2021				Third pandemic wave 18 March, 2021 – 6 May, 2021				Fourth pandemic wave 31 December, 2021 – 9 February, 2022			
	Uppsala city		Uppsala county		Uppsala city		Uppsala county		Uppsala city		Uppsala county	
	Highest TRR (95% CI)	Lowest TRR (95% CI)	Highest TRR (95% CI)	Lowest TRR (95% CI)	Highest TRR (95% CI)	Lowest TRR (95% CI)	Highest TRR (95% CI)	Lowest TRR (95% CI)	Highest TRR (95% CI)	Lowest TRR (95% CI)	Highest TRR (95% CI)	Lowest TRR (95% CI)
Women, ages 5-14	0.65 (0.57, 0.73)	0.60 (0.51, 0.72)	0.61 (0.52, 0.71)	0.60 (0.50, 0.72)	0.64 (0.57, 0.71)	0.60 (0.54, 0.67)	0.60 (0.52, 0.69)	0.58 (0.51, 0.67)	0.75 (0.65, 0.86)	0.65 (0.59, 0.72)	0.61 (0.49, 0.76)	0.57 (0.49, 0.67)
Men, ages 5-14	0.60 (0.52, 0.69)	0.54 (0.45, 0.65)	0.69 (0.58, 0.82)	0.65 (0.51, 0.82)	0.63 (0.57, 0.71)	0.61 (0.55, 0.67)	0.68 (0.60, 0.76)	0.65 (0.58, 0.72)	0.77 (0.69, 0.86)	0.65 (0.59, 0.71)	0.63 (0.52, 0.76)	0.58 (0.50, 0.68)
Women, ages 15-29	0.83 (0.78, 0.88)	0.80 (0.73, 0.87)	1.06 (0.97, 1.17)	0.98 (0.91, 1.04)	0.85 (0.80, 0.90)	0.83 (0.78, 0.89)	0.89 (0.83, 0.95)	0.87 (0.82, 0.94)	1.02 (0.91, 1.15)	0.91 (0.84, 1.00)	0.93 (0.84, 1.03)	0.91 (0.78, 1.06)
Men, ages 15-29	0.84 (0.79, 0.90)	0.82 (0.75, 0.90)	0.98 (0.89, 1.07)	0.94 (0.87, 1.01)	0.84 (0.78, 0.91)	0.82 (0.76, 0.89)	0.89 (0.82, 0.96)	0.86 (0.80, 0.93)	0.99 (0.88, 1.11)	0.89 (0.81, 0.98)	0.87 (0.73, 1.03)	0.86 (0.77, 0.96)
Women, ages 30-49	0.75 (0.71, 0.79)	0.72 (0.68, 0.76)	0.77 (0.73, 0.82)	0.76 (0.71, 0.81)	0.79 (0.75, 0.84)	0.78 (0.74, 0.83)	0.79 (0.75, 0.84)	0.79 (0.75, 0.83)	0.95 (0.87, 1.04)	0.86 (0.79, 0.93)	0.79 (0.69, 0.92)	0.78 (0.71, 0.85)
Men, ages 30-49	0.74 (0.69, 0.79)	0.72 (0.67, 0.78)	0.78 (0.73, 0.83)	0.76 (0.71, 0.82)	0.76 (0.71, 0.82)	0.75 (0.70, 0.81)	0.79 (0.73, 0.85)	0.78 (0.72, 0.84)	0.88 (0.80, 0.96)	0.80 (0.74, 0.86)	0.75 (0.64, 0.89)	0.74 (0.65, 0.83)
Women, ages 50-69	0.90 (0.85, 0.96)	0.87 (0.81, 0.93)	0.95 (0.89, 1.02)	0.91 (0.84, 1.00)	0.94 (0.89, 0.99)	0.92 (0.87, 0.97)	0.97 (0.91, 1.05)	0.95 (0.88, 1.02)	1.07 (0.96, 1.20)	0.97 (0.90, 1.05)	1.02 (0.89, 1.16)	0.93 (0.85, 1.03)

Men, ages 50-69	0.87 (0.82, 0.92)	0.84 (0.79, 0.90)	0.96 (0.87, 1.05)	0.94 (0.87, 1.03)	0.89 (0.83, 0.96)	0.87 (0.81, 0.94)	0.94 (0.83, 1.06)	0.90 (0.80, 1.01)	0.99 (0.88, 1.12)	0.90 (0.82, 0.98)	1.00 (0.86, 1.17)	0.90 (0.79, 1.03)
Women, ages 70+	1.20 (1.04, 1.38)	1.17 (1.00, 1.36)	1.39 (1.08, 1.80)	1.34 (1.08, 1.66)	1.12 (0.95, 1.32)	1.02 (0.87, 1.19)	1.16 (0.99, 1.36)	1.06 (0.92, 1.23)	1.20 (0.88, 1.63)	1.00 (0.80, 1.26)	1.21 (0.93, 1.57)	1.10 (0.89, 1.35)
Men, ages 70+	1.18 (1.02, 1.35)	1.12 (0.95, 1.32)	1.27 (1.05, 1.53)	1.23 (1.07, 1.42)	1.17 (0.98, 1.40)	1.09 (0.93, 1.29)	1.12 (0.98, 1.28)	1.01 (0.89, 1.15)	0.96 (0.69, 1.33)	0.88 (0.71, 1.10)	1.26 (0.98, 1.63)	1.06 (0.85, 1.31)

Supplementary Table 5. Highest and lowest test rate ratios (TRRs) with 95 confidence intervals (CIs) for distance to nearest main testing station (**per 1 kilometre**) presented per sex and age groups in Uppsala County and Uppsala City for the earlier part of the study period (24 June 2020–11 October 2020). The model is adjusted for Care Need Index (CNI), date, day of week of test, age group, sex, Uppsala County/Uppsala City, and daily case notification rates per age and sex group per 100 000 per postal code area. In the youngest age groups (9-14 years), testing data were available from August 1.

	Uppsala County		Uppsala City	
	Highest TRR (95% CI)	Lowest TRR (95% CI)	Highest TRR (95% CI)	Lowest TRR (95% CI)
Women, ages 9-14	0.981 (0.968, 0.994)	0.975 (0.958, 0.992)	0.958 (0.925, 0.993)	0.940 (0.916, 0.964)
Men, ages 9-14	0.987 (0.976, 0.998)	0.969 (0.955, 0.983)	0.958 (0.928, 0.990)	0.949 (0.909, 0.990)
Women, ages 15-29	0.990 (0.983, 0.996)	0.980 (0.970, 0.990)	0.981 (0.963, 1.000)	0.970 (0.949, 0.990)
Men, ages 15-29	0.986 (0.979, 0.993)	0.984 (0.973, 0.995)	0.987 (0.970, 1.005)	0.963 (0.937, 0.990)
Women, ages 30-49	0.995 (0.991, 0.999)	0.984 (0.978, 0.990)	1.000 (0.990, 1.011)	0.997 (0.984, 1.010)
Men, ages 30-49	0.995 (0.990, 1.001)	0.981 (0.972, 0.990)	0.993 (0.981, 1.006)	0.976 (0.962, 0.990)
Women, ages 50-69	0.994 (0.989, 0.998)	0.984 (0.978, 0.990)	0.998 (0.980, 1.016)	0.983 (0.967, 1.000)
Men, ages 50-69	0.996 (0.986, 1.007)	0.988 (0.981, 0.996)	0.992 (0.970, 1.014)	0.979 (0.961, 0.998)
Women, ages 70+	1.006 (0.988, 1.023)	0.994 (0.980, 1.008)	1.001 (0.955, 1.049)	0.934 (0.867, 1.007)
Men, ages 70+	1.006 (0.986, 1.026)	0.984 (0.968, 1.000)	0.984 (0.925, 1.046)	0.966 (0.920, 1.013)

Supplementary Table 6. Highest and lowest test rate ratios (TRRs) with 95 confidence intervals (CIs) for distance to nearest main testing station (**per 10 kilometres**) presented per sex and age groups in Uppsala County and Uppsala City for the earlier part of the study period (24 June 2020–11 October 2020). The model is adjusted for Care Need Index (CNI), date, day of week of test, age group, sex, Uppsala County/Uppsala City, and daily case notification rates per age and sex group per 100 000 per postal code area. In the youngest age groups (9-14 years), testing data were available from August 1.

	Uppsala county		Uppsala city	
	Highest TRR (95% CI)	Lowest TRR (95% CI)	Highest TRR (95% CI)	Lowest TRR (95% CI)
Women, ages 9-14	0.824 (0.724, 0.938)	0.773 (0.650, 0.918)	0.651 (0.457, 0.928)	0.536 (0.415, 0.692)
Men, ages 9-14	0.876 (0.782, 0.981)	0.730 (0.630, 0.845)	0.653 (0.471, 0.906)	0.590 (0.385, 0.905)
Women, ages 15-29	0.901 (0.847, 0.959)	0.815 (0.735, 0.904)	0.828 (0.684, 1.003)	0.734 (0.594, 0.907)
Men, ages 15-29	0.871 (0.809, 0.937)	0.852 (0.761, 0.954)	0.880 (0.738, 1.050)	0.686 (0.521, 0.902)
Women, ages 30-49	0.950 (0.910, 0.992)	0.848 (0.797, 0.903)	1.005 (0.908, 1.112)	0.969 (0.853, 1.101)
Men, ages 30-49	0.954 (0.902, 1.008)	0.826 (0.754, 0.904)	0.935 (0.826, 1.060)	0.786 (0.682, 0.906)
Women, ages 50-69	0.939 (0.898, 0.982)	0.849 (0.798, 0.905)	0.979 (0.818, 1.172)	0.845 (0.716, 0.998)
Men, ages 50-69	0.962 (0.865, 1.070)	0.889 (0.823, 0.962)	0.920 (0.737, 1.149)	0.809 (0.669, 0.979)
Women, ages 70+	1.058 (0.890, 1.258)	0.939 (0.817, 1.079)	1.010 (0.631, 1.617)	0.507 (0.240, 1.072)
Men, ages 70+	1.060 (0.873, 1.288)	0.852 (0.723, 1.005)	0.850 (0.460, 1.570)	0.705 (0.437, 1.140)

SUPPLEMENTARY FIGURES

Supplementary Figure 1. Placements of the four stations for patient-initiated COVID-19 PCR testing initially set up across Uppsala County in June 2020 - in the north, east, and northwest part of the county, and in the centre of Uppsala City.

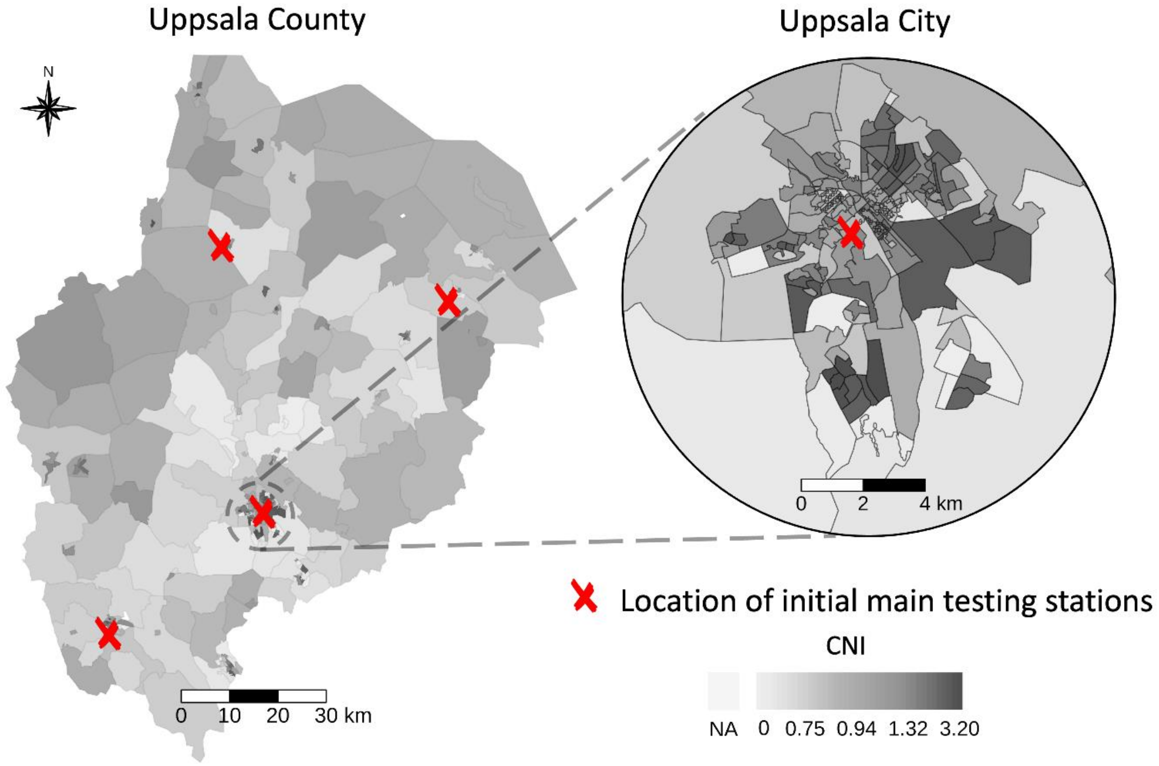

Supplementary Figure 2. The frequency distribution of postal code areas per Care Need Index (CNI) in Uppsala County (n=203) and Uppsala City (n=147). Higher CNI indicates higher primary health care burden.

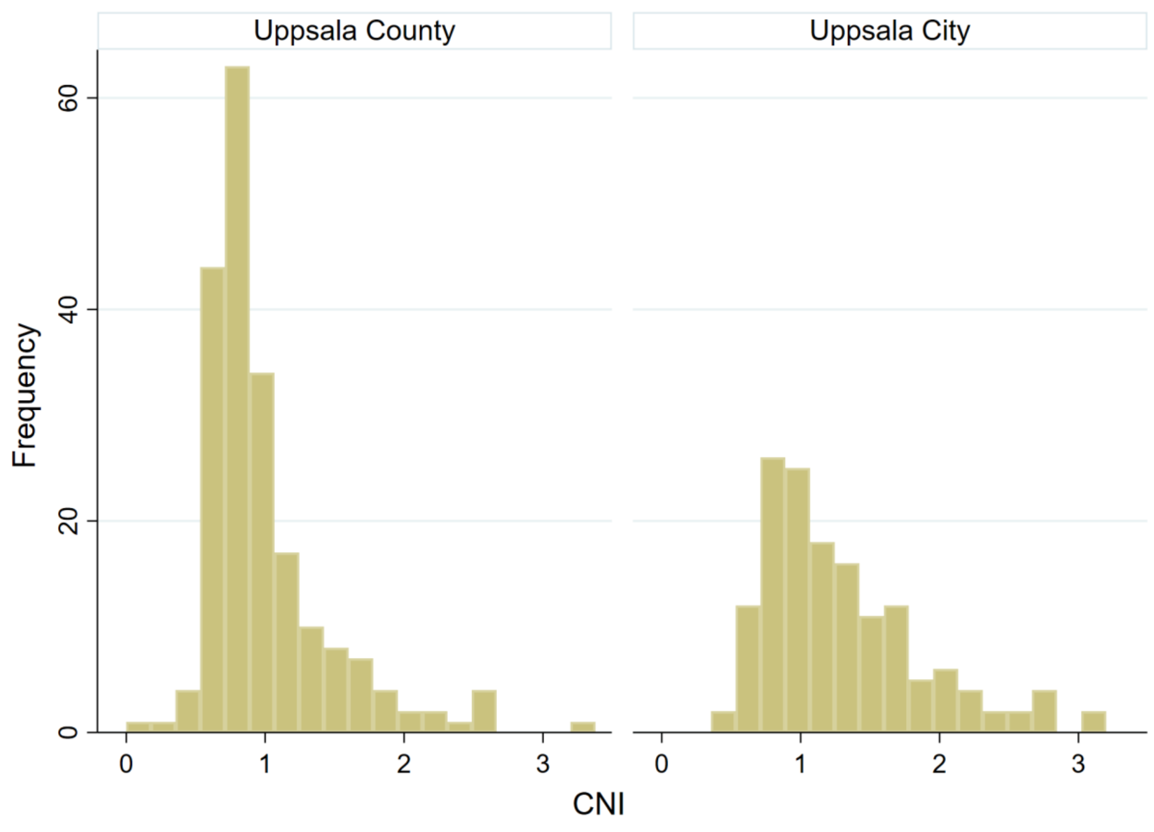

Supplementary Figure 3. Population-weighted Pearson correlation matrices for the composite measure Care Need Index (CNI), proportion of women (%), the seven sociodemographic variables included in CNI*, and distance to nearest main testing station (only applicable 24 June to October 11, 2020) in Uppsala County (left) and Uppsala City (right).

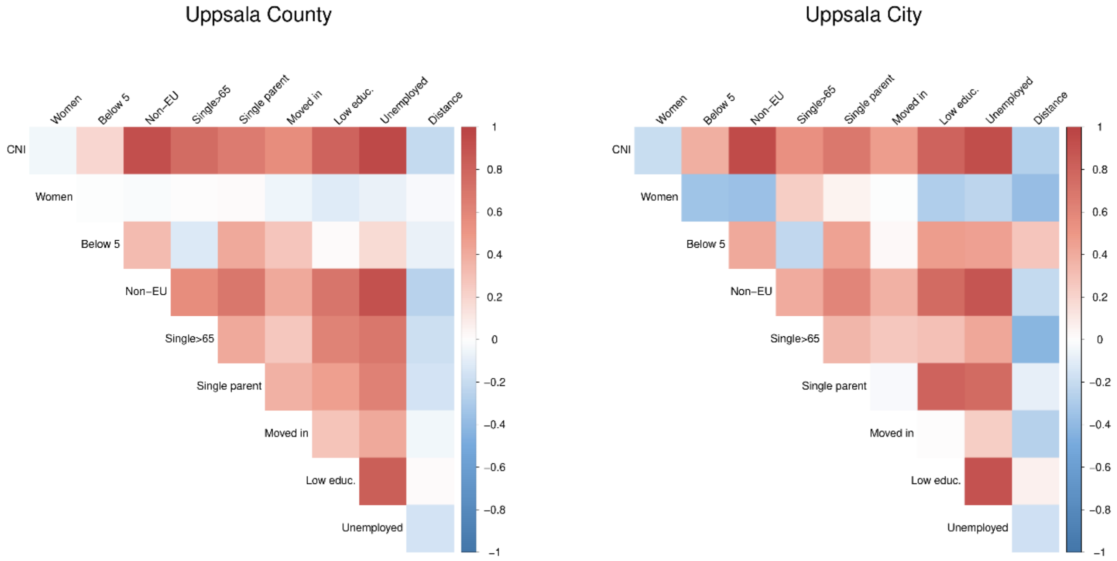

*Proportion of inhabitants in a postal code area who were 1) <5 years, 2) born in East Europe (outside the European Union), Asia, Africa, or South America, 3) >65 years and reside in single-person households, 4) single parents with children <18 years, 5) >1 years and have moved into the postal code area within the previous calendar year, 6) 25–64 years with low educational attainment (≤9 years of schooling, equivalent to compulsory education only in Sweden), 7) 16–64 years and unemployed or enrolled in a labour market programme.

Supplementary Figure 4. 7-day moving averages of daily number of COVID-19 PCR tests, positive tests and hospital admissions per 100 000 inhabitants across the study period (24 June 2020–9 February 2022) for inhabitants residing postal code areas in Uppsala County or Uppsala City.

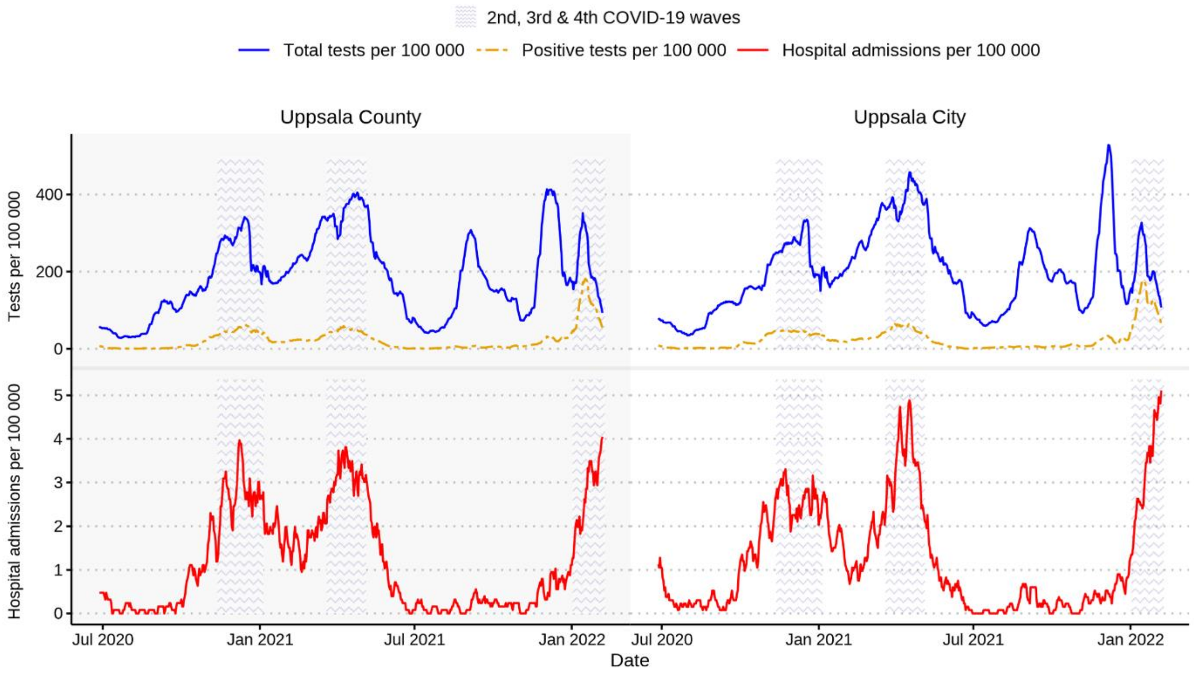

Supplementary Figure 5. 7-day moving averages of daily number of COVID-19 PCR tests and positive tests per sex and age group per 100 000 inhabitants, across the study period (24 June 2020–9 February 2022) for inhabitants residing in a postal code area in Uppsala County (a) and Uppsala City (b).

a)

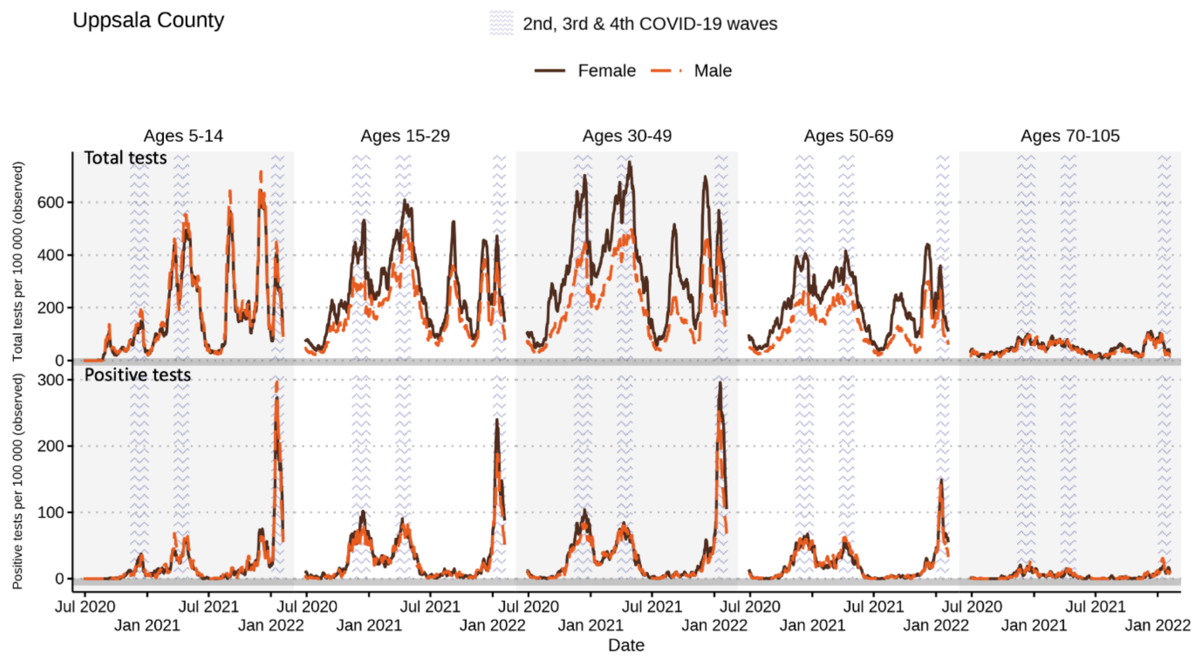

b)

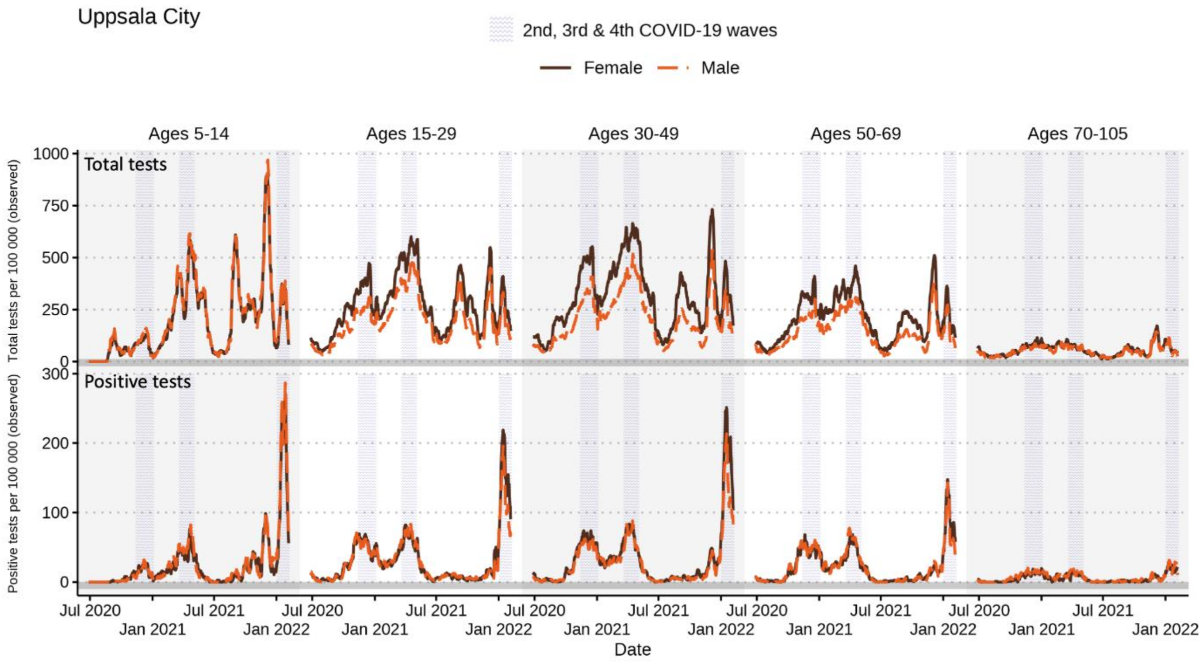

Supplementary Figure 6. Population-weighted cumulative COVID-19 vaccination coverage (defined as ≥2 doses) in inhabitants 15-105 years in Uppsala County (left) and Uppsala City (right), by quartiles of Care Need Index (CNI; Q1 represents lowest CNI quartile). Only vaccinations administrated at healthcare units within Uppsala County Council are included in these data.

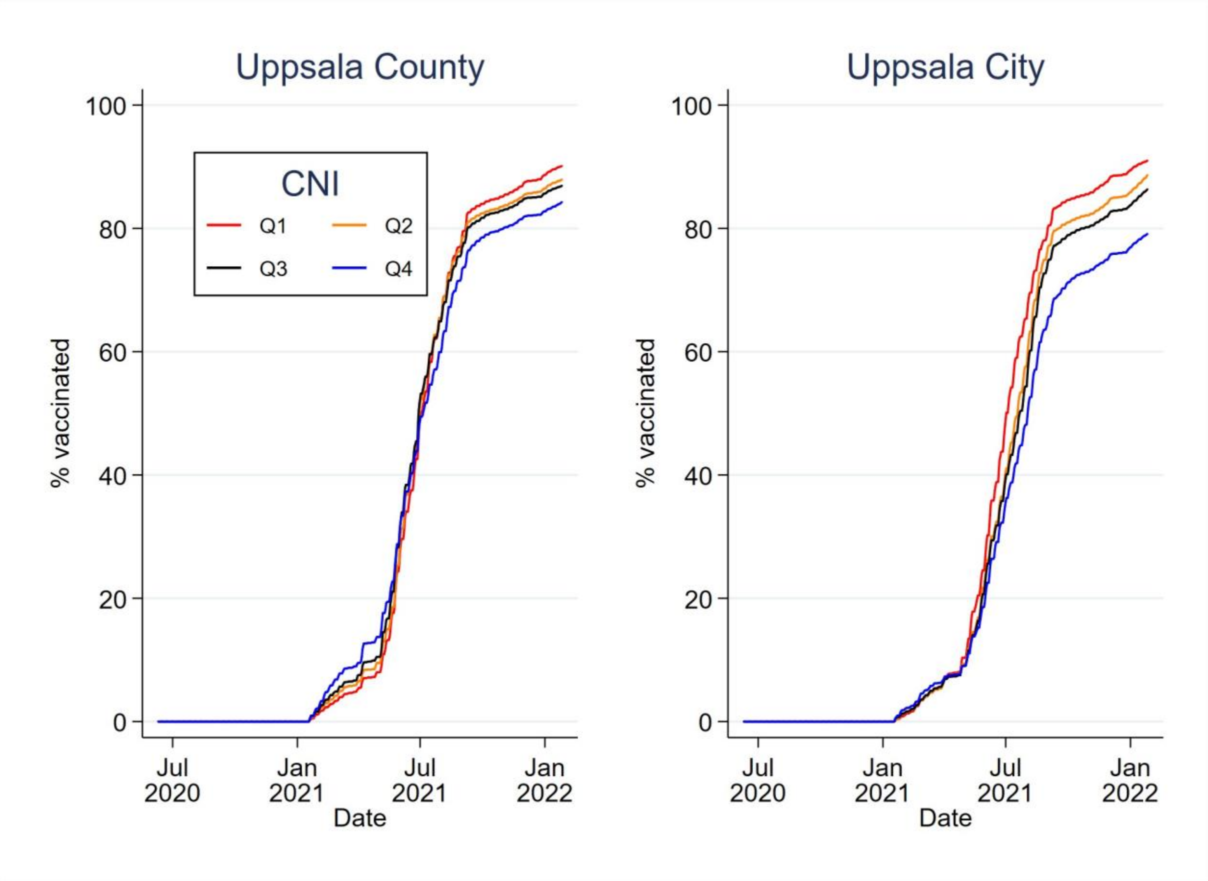

Supplementary Figure 7. 7-day moving averages of daily number of COVID-19 PCR tests per sex and age group per 100 000 inhabitants residing in postal code areas with CNI within the first and fifth CNI quintiles, respectively, in Uppsala County (a) and Uppsala City (b). These moving averages are not model-based and thereby not adjusted for any marker of community transmission (case notification rates or hospital admission rates).

a)

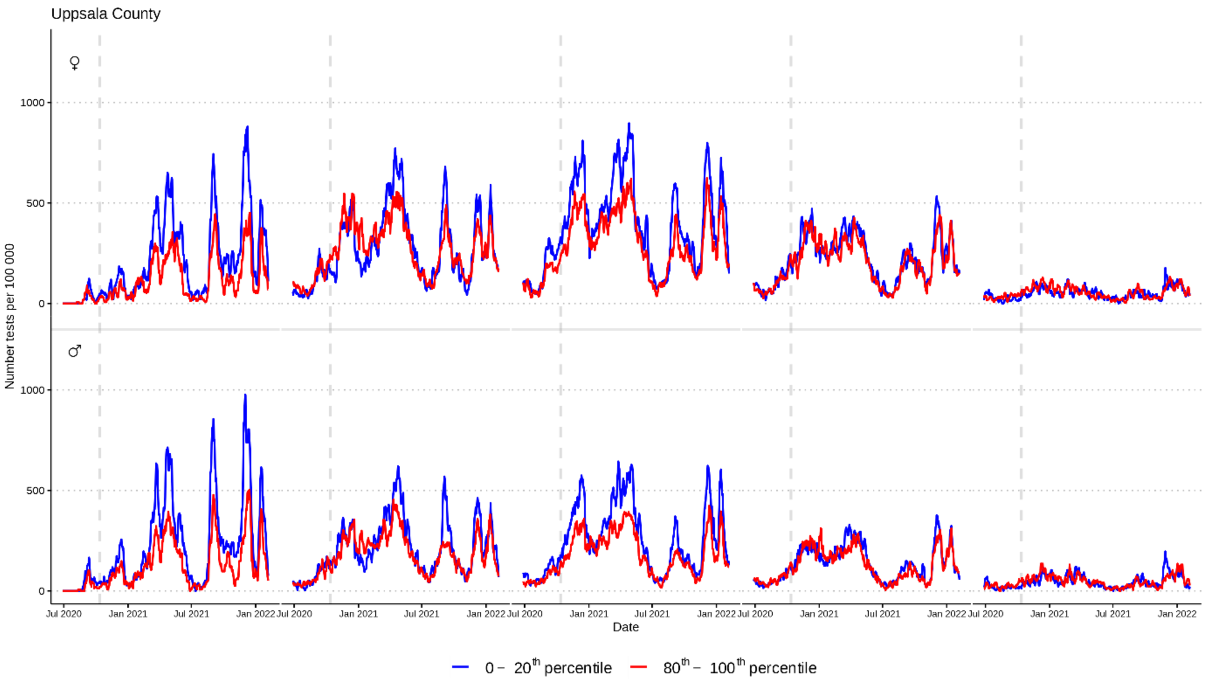

b)

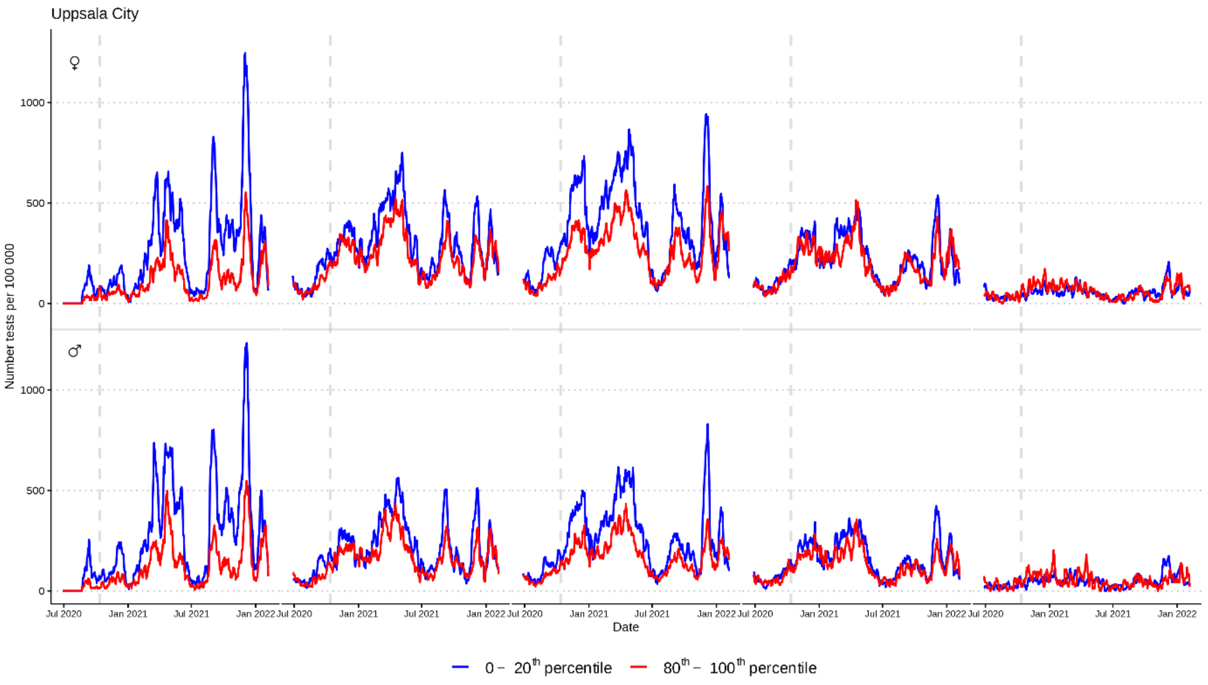

Supplementary Figure 8. Sensitivity analysis. Model-based testing rates for COVID-19 per sex and age group per 100 000 inhabitants in Uppsala County (not including Uppsala City) (a) and Uppsala City (b) across the study period (24 June 2020–9 February 2022), presented for the 10th and 90th postal code area for the Care Need Index (CNI) percentiles. Models are adjusted for number of daily COVID-19 hospital admissions per 100 000 inhabitants.

a)

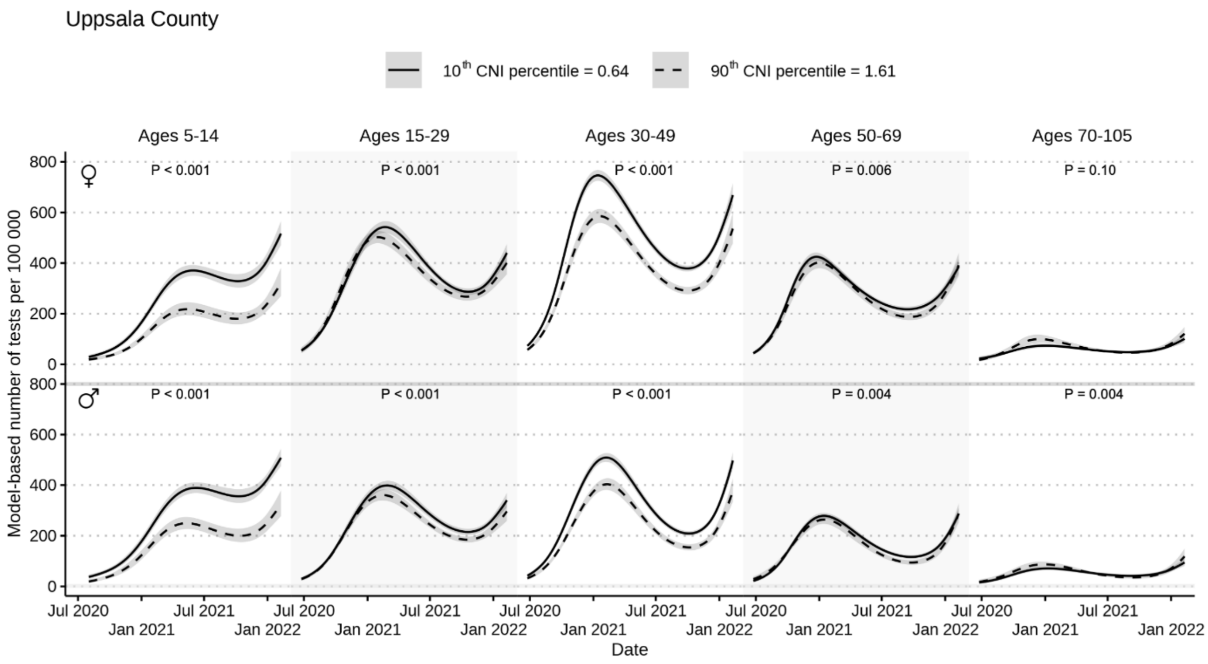

b)

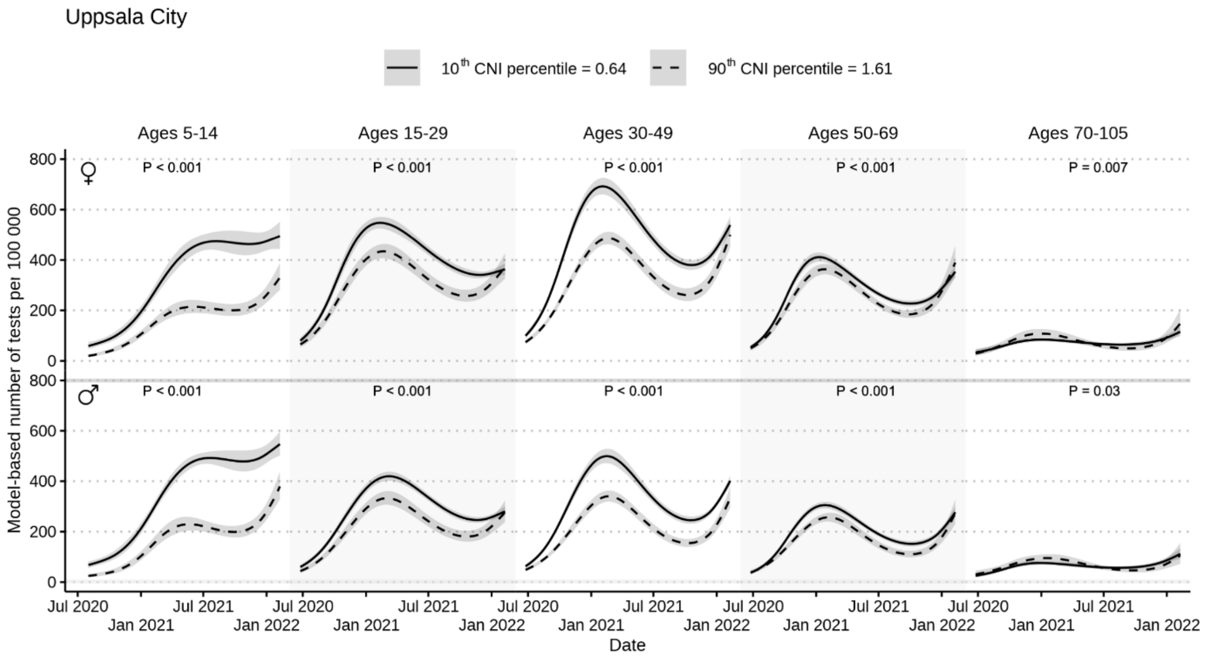

Supplementary Figure 9. Model based testing rates for COVID-19 per sex and age group per 100 000 inhabitants in Uppsala County (not including Uppsala City) (a) and Uppsala City (b) across the early part of the study period (24 June 24–11 October 2020), presented for the 10th and 90th postal code area for the distance to nearest main testing station (in kilometres).

a)

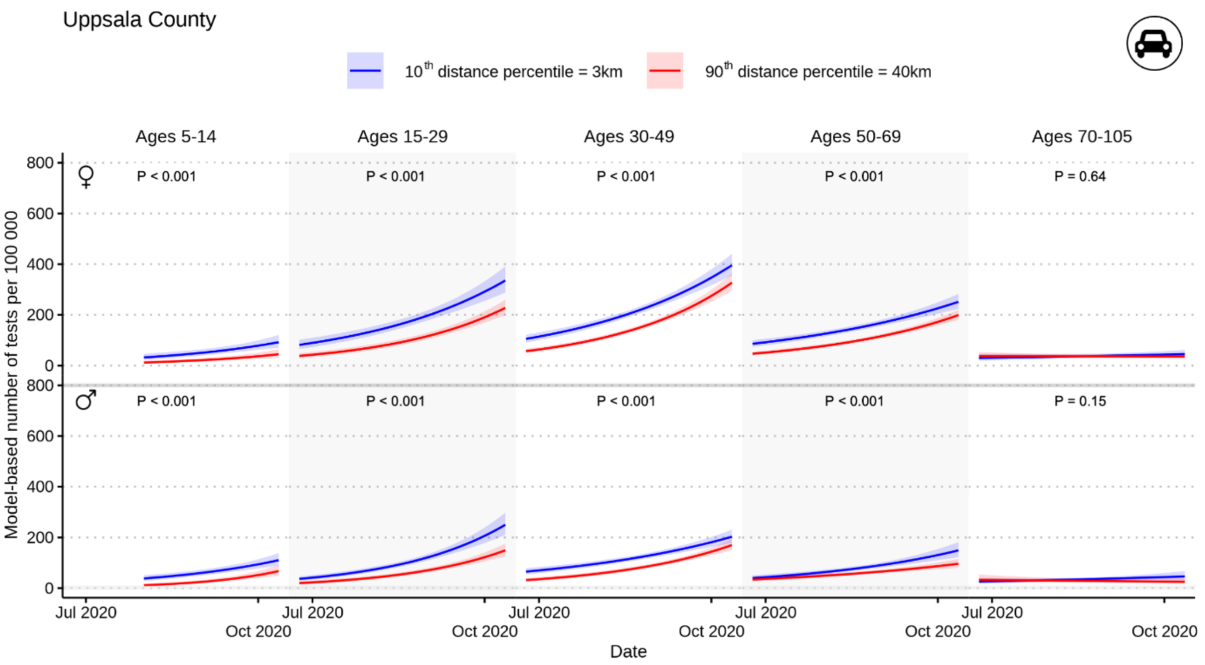

b)

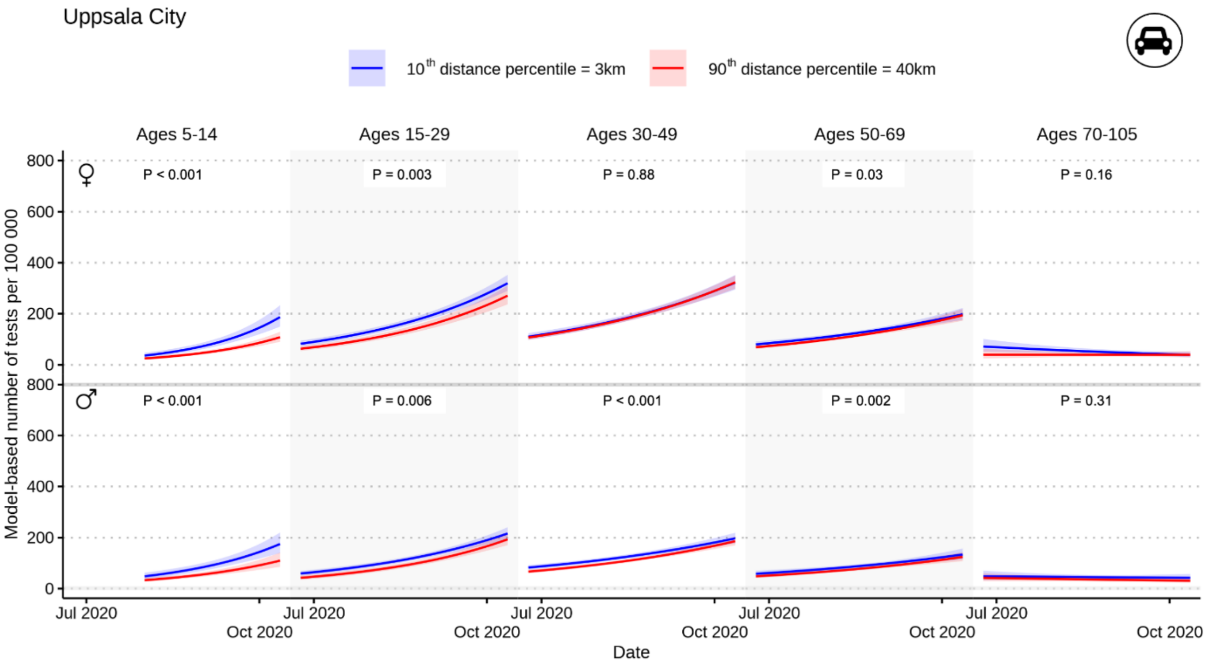

Supplementary Figure 10. 7-day moving averages of daily number of COVID-19 tests and positive tests per 100,000 in inhabitants residing in Gottsunda or Sävja from July 14, 2020 to January 10, 2021.

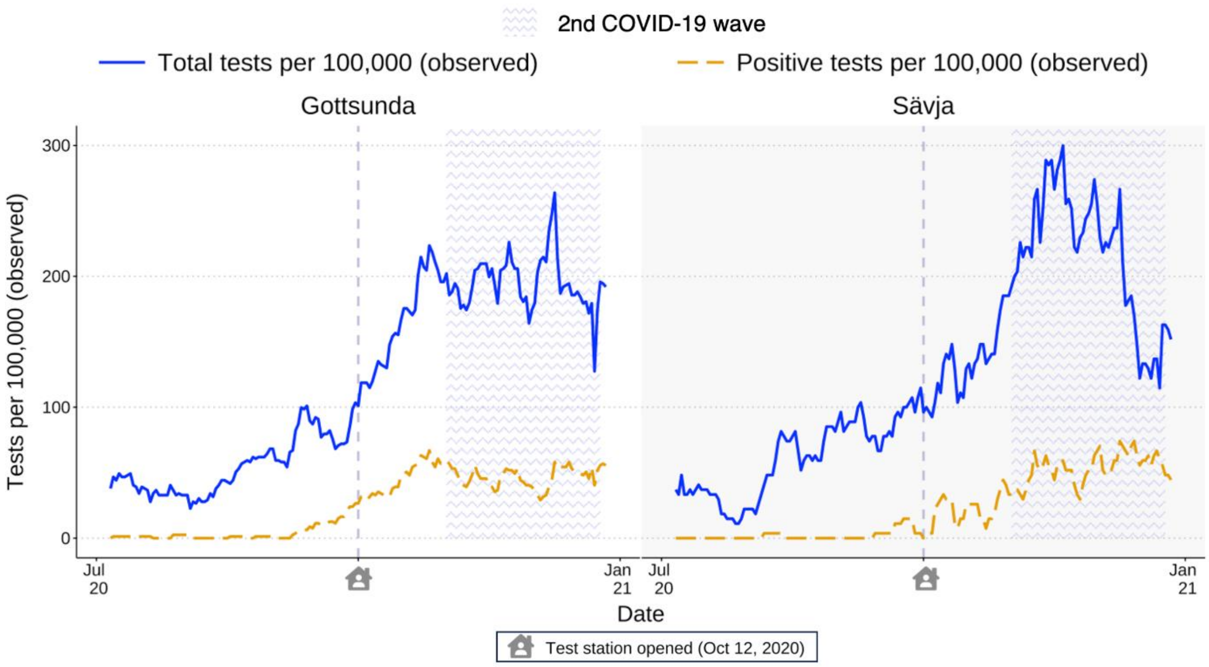

Supplementary Figure 11. 7-day moving averages (MA) of daily number of COVID-19 tests per 100,000 per age group in inhabitants residing in Gottsunda or Sävja from July 14, 2020 to January 10, 2021.

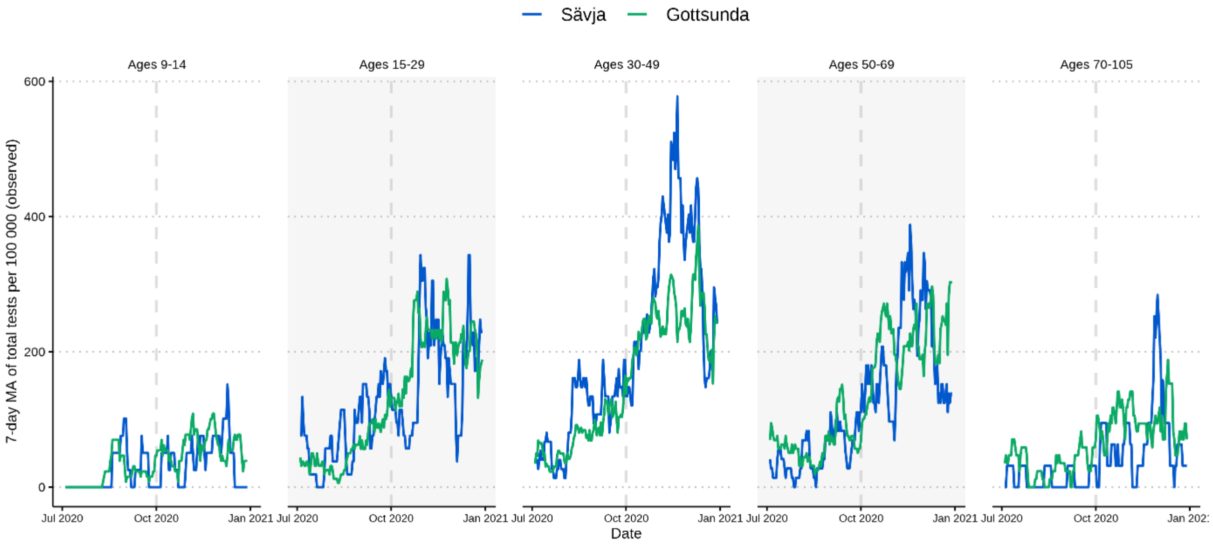

REFERENCES

1. Statistics Sweden. Care Need Index (CNI) 2022 [Available from: https://www.scb.se/vara-tjanster/bestall-data-och-statistik/regionala-statistikprodukter/care-need-index-cni/.
2. Sundquist K, Malmstrom M, Johansson SE, Sundquist J. Care Need Index, a useful tool for the distribution of primary health care resources. J Epidemiol Community Health. 2003;57(5):347-52.
3. Malmstrom M, Sundquist J, Bajekal M, Johansson SE. Indices of need and social deprivation for primary health care. Scand J Soc Med. 1998;26(2):124-30.
4. The Public Health Agency of Sweden. De flesta åtgärder mot covid-19 upphör den 9 februari (Most measures against COVID-19 cease February 9). https://www.folkhalsomyndigheten.se/nyheter-och-press/nyhetsarkiv/2022/februari/de-flesta-atgarder-mot-covid-19-upphor-den-9-februari/2022.
5. Region Uppsala. Vaccination av 12-15-åringar startar 11 oktober (Vaccinations of children aged 12-15 will commence October 11) 2022 [Available from: https://regionuppsala.se/politik-och-paverkan/pressrum/2021/september/vaccination-av-12-15-aringar-startar-11-oktober/.
6. The Swedish Paediatric Society. Rekommendationer för vaccination mot covid-19 för särskilda grupper av barn från 5 års ålder. Uppdaterat 2021-12-16. (Recommendations for COVID-19 vaccinations for selected groups of children ≥5 years. Updated December 16, 2022). https://www.barnlakarforeningen.se/wp-content/uploads/2021/12/BLF-Rekommendationer-for-vaccination-mot-covid-19-for-sarskilda-grupper-av-barn-fran-5-ars-alder-20211216.pdf2021 [
7. Dimick JB, Ryan AM. Methods for evaluating changes in health care policy: the difference-in-differences approach. JAMA. 2014;312(22):2401-2.
